# Protective mechanisms and therapeutic promise of nanomaterials in radiation-induced diseases

**DOI:** 10.1016/j.ijpx.2026.100658

**Published:** 2026-09-03

**Authors:** Yizhi Ge, Minghong Xu, Xingyu Yang, Wen Yun, Wenxuan Huang, Jia Liu

**Affiliations:** a Department of Radiation Oncology, Jiangsu Cancer Hospital & Jiangsu Institute of Cancer Research & The Affiliated Cancer Hospital of Nanjing Medical University, Nanjing, Jiangsu 210009, China; b Hubei Key Laboratory of Industry Microbiology, National “111” Center for Cellular Regulation and Molecular Pharmaceutics, Glyn O. Phillips Hydrocolloid Research Centre at HBUT, School of Life and Health Sciences, Hubei University of Technology, Wuhan 430068, China; c Department of General Surgery, Jiangsu Cancer Hospital & Jiangsu Institute of Cancer Research & The Affiliated Cancer Hospital of Nanjing Medical University, Nanjing, Jiangsu 210009, China

**Keywords:** Reactive oxygen species, Targeted drug delivery, Nanozymes, Normal-tissue selectivity, Good health and well-being

## Abstract

Radiotherapy is one of the common approaches for tumor treatment by suppressing tumor growth, which, however, inevitably leads to severe damage to surrounding healthy tissues. Although significant advances have been made in the development of radioprotective agents, the high toxicity and low bioavailability are still the main obstacles to their applications. Recently, the emergence of nanotechnology has brought promising solutions to address these challenges. Owing to their unique physicochemical properties, such as precisely controllable size, functionalized surfaces, excellent biocompatibility, and efficient drug delivery capabilities, nanomaterials have shown great potential in revolutionizing radiation protection medicine. This review systematically summarizes the developments of nanomaterial-based radioprotection, focusing on the protective mechanism, design innovation, and practical applications. It is expected that this review could provide researchers with a comprehensive understanding of the progress in nanomaterial-mediated radioprotection, thereby generating novel inspiration and approaches to facilitate the development of effective nano-therapeutics for radiation-related diseases.

## Highlights

• Radiotherapy is a major approach to cancer treatment, but off-target radiation can significantly damage healthy tissues.
• Nanomaterials are promising radioprotective candidates, offering new ways to prevent or alleviate radiation damage.
• This review covers recent advances in nanomaterial-based radioprotection, focusing on mechanisms, design, and applications.
• Future work includes AI-assisted nanomaterial design, long-term biosafety, mechanisms, and optimization of approved agents.

## Graphical abstract

**Figure:**
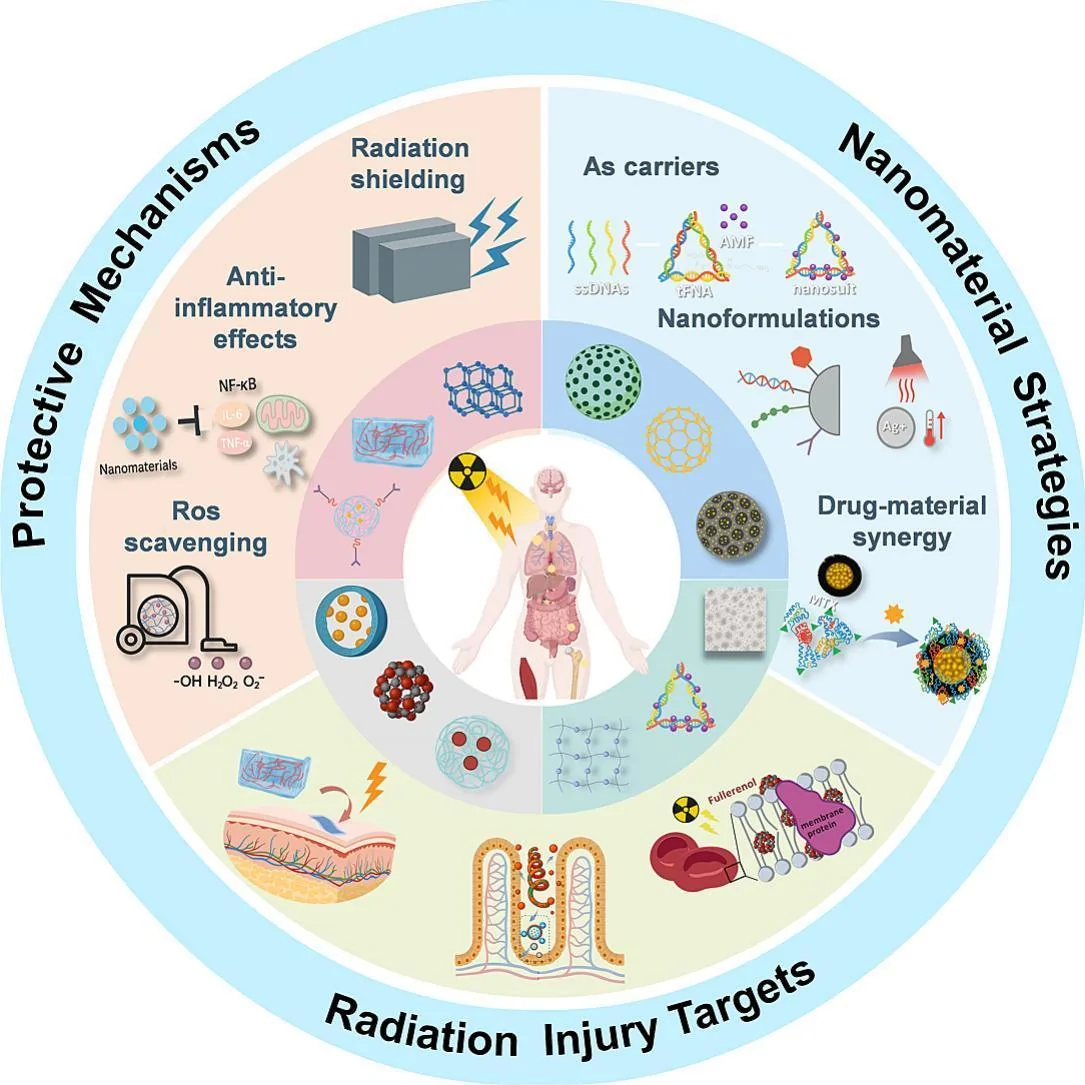

## 1 Introduction

Radiotherapy, one of the important approaches in cancer treatment, employs high-energy ionizing radiation to exert comprehensive antitumor effects by directly damaging tumor DNA through ionization and indirectly inducing oxidative stress via reactive oxygen species (ROS) generation. Furthermore, radiotherapy disrupts the tumor vasculature and modulates the immunosuppressive tumor microenvironment, thereby enhancing antitumor effects. Radiotherapy is utilized in over 50% of cancer patients, with 40% of cases achieving significant tumor suppression or even complete remission (Lilley and Murray, 2023; De Ruysscher et al., 2019). However, neither whole-body nor localized radiation treatments can achieve targeted cancer therapy at single-cell resolution. Thus, radiotherapy inevitably causes damage to the surrounding healthy tissues, resulting in a series of side effects and complications (Farhood et al., 2020). Similar to the effects on tumor cells, high-energy radiation (such as X-rays and γ-rays) can induce DNA damage in healthy cells, causing severe single- or double-strand DNA breaks. In addition, radiation interacts with water molecules to produce ROS, such as superoxide radical anions (O_2_^·-^), hydrogen peroxide (H_2_O_2_), and hydroxyl radicals (·OH). These reactive species further destroy cellular macromolecules, leading to cell dysfunction and death (Ibáñez et al., 2024; Hasegawa et al., 2020). Through these direct and indirect mechanisms, radiotherapy causes cytotoxic effects on healthy tissues, leading to organ damage and dysfunction. The skin, as the body's first defense barrier, shows high sensitivity to radiation exposure, and about 95% of patients experience acute skin injury after receiving radiotherapy (Kišonas et al., 2021). Erythema, blisters, and ulcers are common symptoms of radiation-induced skin injury (RISI), often accompanied by pain and pruritus. The gastrointestinal tract ranks as the second most radiosensitive organ system. Radiation-induced gastrointestinal injury (RIGI) usually occurs in patients receiving radiotherapy for abdominal and pelvic malignancies, characterized by abdominal pain, diarrhea, bloody stool, and other complications (Chen et al., 2021; Liu et al., 2025). Specifically, lung injury is a major complication in patients receiving radiotherapy for thoracic malignancies, including lung cancer, esophageal cancer, and breast cancer (Zareie et al., 2022; Hanania et al., 2019). In addition, radiotherapy causes radiation-induced hematopoietic injury (RIHI), damaging hematopoietic stem/progenitor cells and leading to myelosuppression and cytopenia (Zhang et al., 2024). These radiotherapy-induced injuries to healthy tissues exacerbate the already poor survival rates and quality of life in cancer patients, and greatly reduce treatment compliance, representing an urgent and critical challenge in cancer therapy.

In this context, the development of effective radioprotective agents is of paramount importance for cancer treatment as well as for the prevention and treatment of various diseases induced by ionizing radiation. Rather than relying only on their general physicochemical properties, nanomaterials offer particular advantages as programmable delivery and therapeutic platforms for radioprotection. Properly designed, nanomaterial-based delivery systems can increase drug stability and in-body circulation time, thereby improving drug bioavailability and radiological protection effects (Pandey et al., 2025). Nanocarriers can also achieve passive accumulation through the enhanced permeability and retention (EPR) effect or active targeting through surface modification with tissue- or cell-specific ligands, thereby increasing drug exposure in tissues requiring protection while reducing off-target toxicity. In addition, stimuli-responsive nanocarriers can release their cargos in response to pathological signals, such as acidic pH, elevated ROS levels, enzymes, or radiation itself, enabling sustained or on-demand drug release (Kobzev et al., 2026). These targeting and controlled-release properties may improve the spatial and temporal precision of radioprotective interventions. For instance, some nanomaterials (e.g., C_60_ fullerene nanoparticles) have free radical scavenging ability to protect healthy tissues from radiation-induced oxidative damage (Xie et al., 2019a). In summary, nanomaterial-based radioprotection may simultaneously improve drug delivery, tissue selectivity, and intrinsic protection against the adverse effects of cancer radiotherapy.

**Scheme 1:**
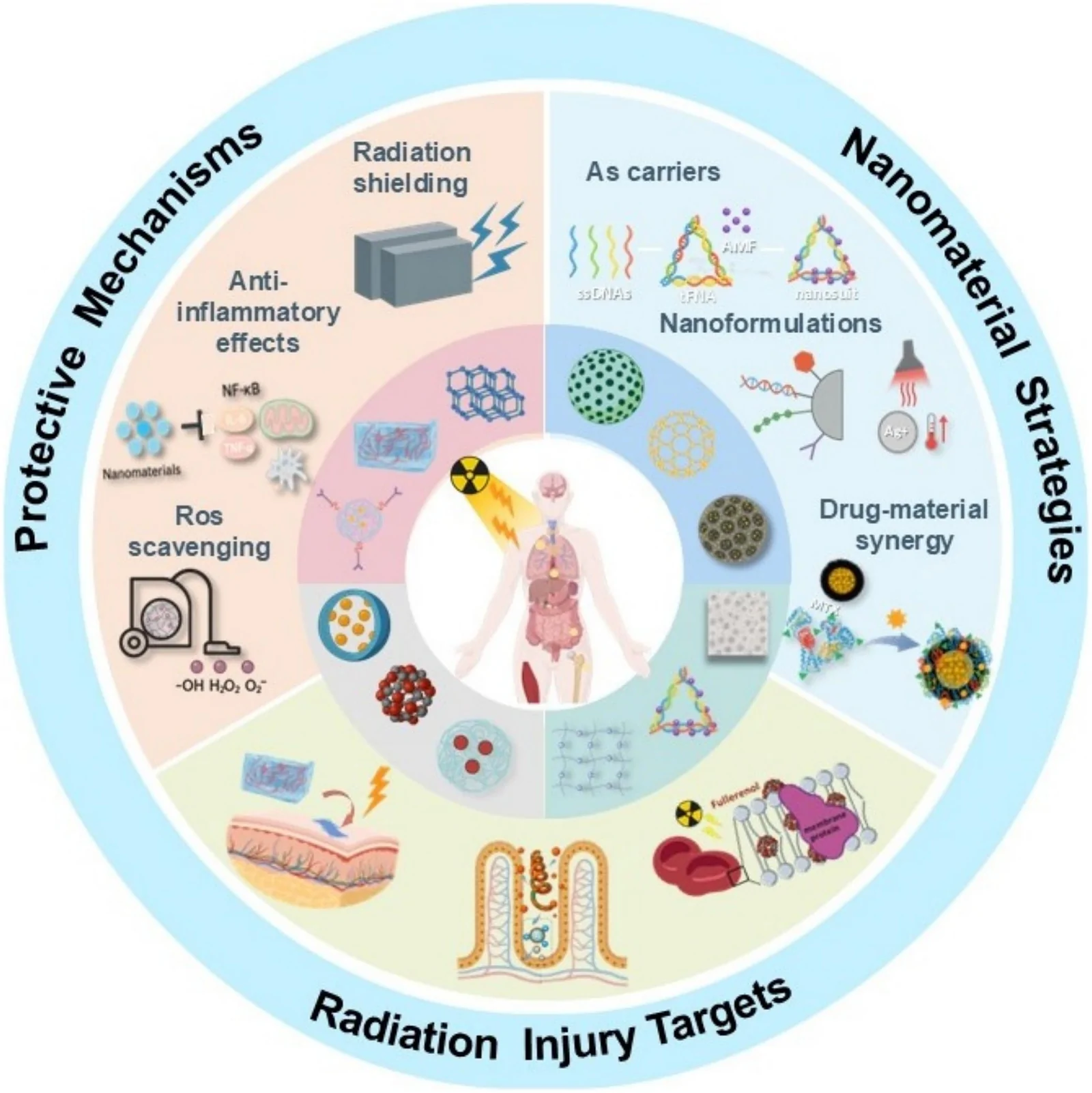
The protective mechanisms and therapeutic strategies of nanomaterials for the treatment of radiation-induced injury.

In this review, we highlight and summarize the research advances in multifunctional nanomaterials for radiation protection (Scheme 1). A mechanism–platform–disease framework, which connects radioprotective mechanisms with material functions, tissue selectivity, disease-specific applications, and translational considerations was established. Firstly, we discuss the radioprotective ways of nanomaterials, including radiation shielding, ROS elimination, and anti-inflammatory effects. Subsequently, we categorize nanomaterials according to their functional roles in radiation protection, involving nanocarriers for drug delivery, radioprotective nanoagents, and synergies between drugs and nanomaterials. Furthermore, we elucidate the protective mechanisms and effects of nanomaterials in the common radiation-induced diseases. By introducing the comprehensive progress, we aim to help researchers gain a deeper and more comprehensive understanding of nanomaterials in radiation protection, stimulating innovative and novel strategies for radiation-related diseases.

## 2 Rationale for radiation protection

### 2.1 Radiation shielding

Shielding protection refers to the utilization of materials with shielding protective function placed between the radiation source and the irradiated object to absorb and weaken the absorbed dose. Normally, when tumors receive radiation from a particle accelerator, both the tissue and the accelerator's dose scatter randomly, leading to the radiotherapy rays reaching the tissue outside the targets. This is the main cause of radiation damage to healthy tissues (Uddin et al., 2024). Based on this fact, the shielding protection is to shield the radiation of rays to the surrounding tissues by suitable materials with appropriate shapes. Current cancer radiotherapy employs various particle beams generated by accelerators, including photon beams, electron beams, heavy ion beams, proton beams, and neutron rays. Metal and metal oxide nanoparticles exhibit significant functional improvements in shielding due to their extremely high surface areas and enhanced surface interactions (Şevik et al., 2023). Therefore, nanomaterials are usually doped into various polymer materials used for radiation shielding to introduce or enhance their functional properties. For instance, polypropylene or vinyl ethyl acetate are impregnated with metal oxide nanofillers to acquire a strong shielding capability aside from their low density, ease of processing, and versatility strong shielding ability. Polymer/nanomaterial composites have been applied in radiation shielding (Joshi et al., 2024).

#### 2.1.1 Shielding nanomaterials for γ-rays

**Fig. 1:**
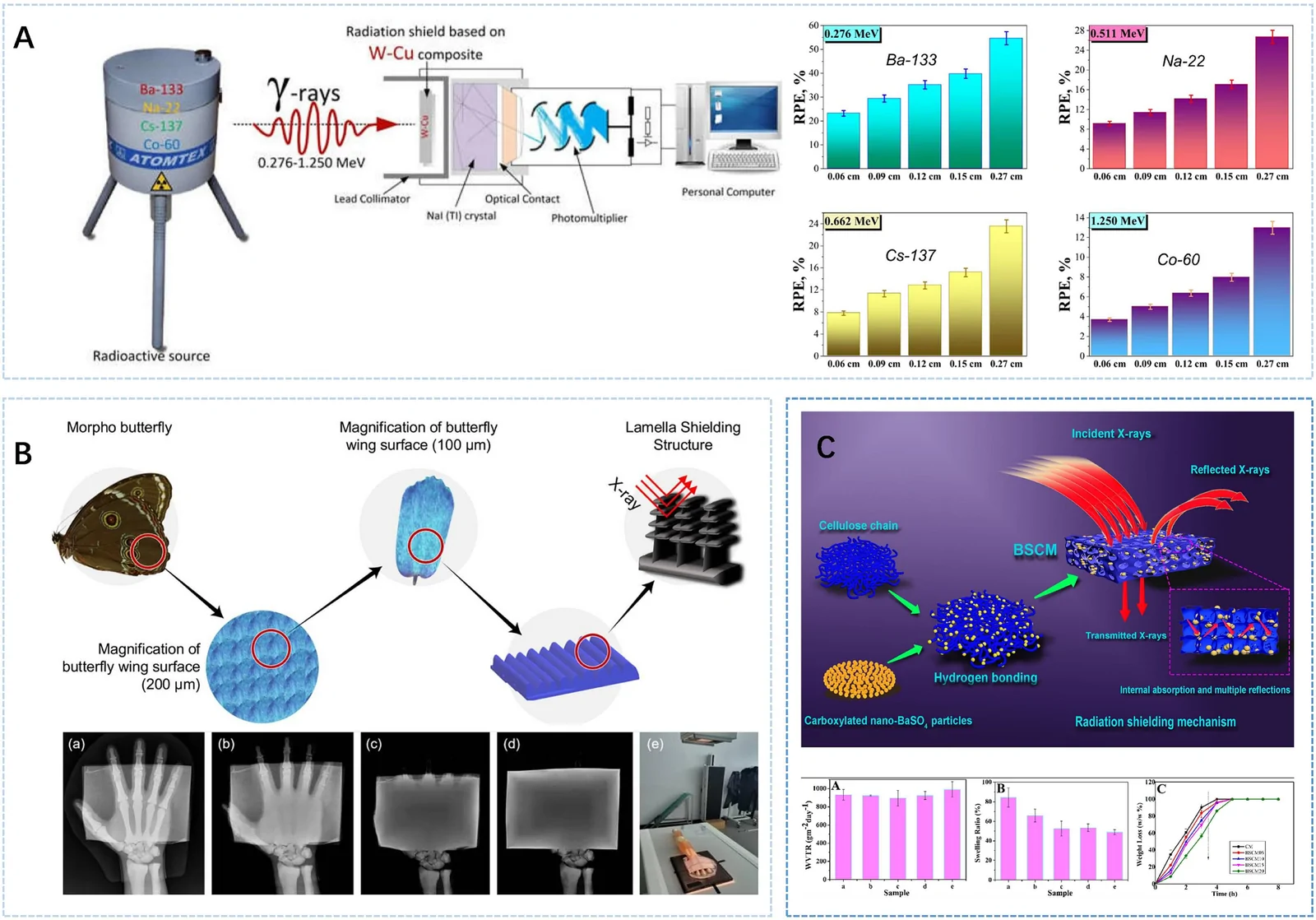
Nanomaterials with radioprotective effects against different types of radiation. A) Composites of tungsten–copper and silicone with nanocrystalline boundaries and high densities demonstrated excellent γ-radiation shielding performance. Reproduced from Ref. (Tishkevich et al., 2022) under the terms of the Creative Commons Attribution (CC BY) 4.0 license. B) Nanofiber papers based on polymer–tungsten composites exhibit good shielding efficiency against X-rays at 60 keV. Reproduced from Ref. (Kim and Byun, 2022) under the terms of the Creative Commons Attribution (CC BY) 4.0 license. C) Carboxylated BaSO_4_ nanoparticles establish hydrogen bonding interactions with cellulose, thereby improving its X-ray shielding performance. Reproduced with permission from Ref. (Jiang et al., 2019) Copyright ©2019 Elsevier Ltd.

γ-rays are produced by the decay of radioactive isotopes (e.g., cobalt-60, iridium-192), which are characterized by concentrated energy (average 1.25 MeV) and strong penetration. Effective shielding materials of γ-rays generally require high atomic numbers and high density, which can absorb or scatter γ-rays through the photoelectric effect, Compton scattering, and the electron pair effect. Nanoferrite is a high-density material with excellent γ-ray shielding capability. Its shielding performance can be tuned by doping with different elements. Doping with Ni or Cu can enhance the radiation shielding effectiveness of iron oxide in the energy range of 122–1330 keV (Gaikwad et al., 2024), while doping with Mg or Zn increases the material's density, thereby improving its shielding efficiency against low-energy gamma rays (Zakaly et al., 2022). Silicone rubber (dimethylpolysiloxane) is an ideal shielding material due to its elasticity, attenuation properties, and cost-effectiveness. Metal oxide nanoparticles can be embedded into silicone rubber to form composite materials to promote the shielding properties. For example, tin oxide and cadmium oxide nanoparticles are able to endow composite materials with enhanced γ-ray protection capability (Gouda and Zard, 2024). The incorporation of nanosized lead oxide significantly increased the linear attenuation coefficient of the silicone rubber composites and improved the radiation protection parameters, especially at low γ energies (El-Khatib et al., 2022). In addition, tungsten‑copper/silicone composites with nanocrystalline boundaries and high densities exhibited high shielding efficiency against γ radiation (Fig. 1A).

#### 2.1.2 Shielding nanomaterials for X-rays

X-rays are produced by linear accelerators (electron bombardment of tungsten targets) or ordinary X-ray machines and can be controlled from low energy (such as for skin treatments) to high energy (such as above 6 MeV for deep tumors) (Dillon et al., 2023). Nano-clays, a class of natural silicate nanoparticles, demonstrate a radiation protection effect, which can be used to shield X-ray radiation. The green clay originating from Adrar in Algerian Sahara showed more robust radiation shielding, compared with Reggan and Timimoune, with radiation attenuation and mass absorption coefficient of 99.8% and 243.4 cm^2^/g, respectively (Sakher et al., 2022). In addition, composite nanomaterials also play an important role in X-ray chemotherapy protection. The lead oxide (PbO) particles were integrated into polystyrene (PS) to form PS/PbO nanocomposites, whose shielding capabilities were regulated by the PbO concentrations and particle sizes (Osman et al., 2023). The inclusion of bismuth trioxide and tungsten trioxide (Bi_2_O_3_ + WO_3_) nanofillers in dimethylpolysiloxane matrix showed better shielding performance against X-rays than other mono- and polymetallic fillers (Asadpour et al., 2023). Polymer‑tungsten composites were spun by electrostatic spinning in order to produce nanofibers with a multilayer film structure. The nanofiber-based paper exhibited X-ray shielding rates of 64.88% at 0.1 mm and 90.10% at 0.3 mm for 60 keV (Fig. 1B). Jiang et al. constructed a series of BaSO_4_/cellulose nanocomposite membranes (BSCM) as X-ray shielding materials. The carboxylated BaSO_4_ nanoparticles form hydrogen bonds with cellulose, enhancing its X-ray shielding capability (Fig. 1C). Nickel oxide nanopowders (15–35 nm) were embedded into EI-403 epoxy resin to fabricate nanocomposites. Therein, 11%-2 mm, 7%-4 mm, and 7%-6 mm composites showed great shielding efficacy of 46.80%, 66.72%, and 64.52%, respectively (Zaroushani et al., 2016). To obtain a dental shield, four different types of nanopowders (Ti, Zr (IV) oxide, Ag, and Co) were mixed into a polymer matrix at a doping value of 8%. The Co-based nanocomposite (0.20 cm) was found to have the highest transmission value (16.05) (Ayyıldız et al., 2015). The incorporation of metallic and metal oxide nanomaterials significantly enhances the radiation shielding properties of the base materials. This improvement allows the fabrication of nanocomposite protective coverings with proper shapes and thicknesses tailored to specific X-ray shielding requirements, thereby broadening their potential for clinical applications.

#### 2.1.3 Shielding nanomaterials for neutron

Neutron radiotherapy emerges as a novel cancer therapeutic approach, which employs high-energy fast neutrons for radiation treatment. Such modality could directly and effectively damage DNA molecules independent of oxygen-related radicals, and is a promising treatment for hypoxic tumors. Although its micron-scale radiation significantly reduces the risk of damage to healthy cells surrounding the tumor compared to X-rays or γ rays, neutrons exhibit pronounced delayed effects on normal tissues and are more likely to cause late-onset, irreversible tissue damage. Moreover, due to their high linear energy properties, neutron beams would induce severe consequences when causing damage to healthy tissues. Specific materials containing hydrogen and boron possess neutron shielding effects through ejection or absorption. Abdous et al. synthesized a bisphenol A-based polybenzooxazine (BA-PBz) and incorporated hexagonal boron nitride to fabricate nanocomposites with great neutron shielding performance (Abdous et al., 2023). In addition, metal-containing nanomaterials also exhibit neutron shielding effects. Fe-based metal nanoparticles were incorporated in polypropylene to enhance the shielding ability, which was positively correlated with the concentrations of metal nanoparticles. Among them, polypropylene-Fe_5_, a sample consisting of 5 mol% Fe, had the highest ∑R value (1.10650 cm^−1^) and the lowest value of the mean free path for fast neutrons (Alshipli et al., 2023).

Since different radiation conditions cause varying degrees of radiation damage, it is essential to design tailored radiation shields based on the radiation type, intensity, and exposure time. To optimize the shielding effects, the development of nanomaterials with excellent shielding effects and good plasticity is the key challenge that requires further research efforts.

### 2.2 Overcoming radiation-induced oxidative stress

For the surrounding tissues exposed to radiation (such as X-rays and gamma rays), water molecules and other intracellular molecules may be ionized or excited to form various ROS (Reactive Oxygen Species), such as superoxide anions, hydroxyl radicals, and hydrogen peroxide. The cytotoxicity caused by the accumulation of these reactive oxygen species (ROS) in the body or within cells leads to tissue damage (Srinivas et al., 2019). To combat these excessive reactive species, diverse nanomaterials have been explored to protect normal tissues/cells from oxidative stress damage, including directly eliminating ROS in tissues/cells, and promoting the endogenous antioxidant system to counteract oxidative stress (Liao et al., 2021).

#### 2.2.1 Nanomaterials for ROS scavenging

Carbon nanomaterials are a class of nanomaterials primarily composed of carbon atoms, including fullerenes, carbon nanotubes, and graphene. These materials possess high mechanical strength, great electrical and thermal conductivity, and unique optical properties, which are widely used in energy, electronics, biomedicine, and other fields. Graphene Oxide (GO) is an oxidized derivative of graphene. Through oxidation reactions, oxygen-containing functional groups such as hydroxyl, epoxy, and carboxyl groups are introduced onto the surface of graphene, enhancing its reactivity. All carbon atoms of GO nanoparticles are exposed, and the open structure of GO allows the carbon atoms on its edges to efficiently trap free radicals. Low concentrations (10 μg/mL) of GO effectively scavenged ROS, reducing the DNA damage and cell death by 48% and 39% in fibroblasts under X-ray exposure, respectively (Qiao et al., 2014). Studies have shown that graphene bovine serum albumin (BSA) nanoparticles enhanced the radical species scavenging efficacy and restored levels of superoxide dismutase (SOD) and malondialdehyde (MDA), thus improving cell survival under ionizing radiation as well as protecting mouse bone marrow DNA (Xie et al., 2019a). In addition, graphodiyne (GDY) demonstrates the function of scavenging free radicals. BSA was decorated on GDY to synthesize nano-GDY-BSA, which was studied on the gastrointestinal radiation protection ability. Notably, nano-GDY-BSA exhibited excellent properties, such as potent free radical scavenging, high chemical stability under gastric acid conditions, prolonged gastrointestinal retention time, and good oral biosafety, making it a promising nano-agent for gastrointestinal radioprotection. In vitro experiments showed that GDY-BSA nanoparticles reduced DNA damage and improved cell viability in irradiated gastrointestinal cells. In vivo experiments in mice showed that GDY-BSA nanoparticles attenuated radiation-induced diarrhea, weight loss, and histopathological damage in the gastrointestinal tract (Xie et al., 2019a). Nano-GDY-loaded sodium hyaluronate (Nano-GDY@SH) hydrogels were used for dermal application, which had the ability to scavenge free radicals for chemical repair (Xie et al., 2022). Fullerenol is another type of carbon-based nanomaterial and plays an important role in radiation protection. In human keratinocytes, fullerenol significantly blocked ROS-induced damage and enhanced cell viability after radiation. In ray-applied animals, fullerenol-loaded hyaluronate hydrogel protected epidermal stem cells and effectively alleviated radiation dermatitis (Zhao et al., 2021). Moreover, nano‑carbon suspension showed high ROS scavenging activity, which effectively abrogated mitochondrial dysfunction and DNA double-strand breaks to protect cells from radiation-induced damage. Thus, it maintained the balance of intestinal flora, thereby alleviating radiation enteritis (Wang et al., 2020a).

Metal nanomaterials have special physical and chemical properties, including magnetic, mechanical, optical, catalytic, and radiation tolerance properties (Sáenz-Trevizo and Hodge, 2020). The thermal transformation of metal-organic frameworks (MOFs) can produce a variety of nanostructured materials, including carbon-based materials, metal oxides, metal sulfides, metal phosphides, and metal carbides. These MOF-derived materials possess characteristics such as high surface area, permanent porosity, and tunable functionalities, which endow them with excellent performance in various fields (Dang et al., 2017). It is shown that inducing surface strains of metallic nanoparticles can enhance their catalytic activity.

**Fig. 2:**
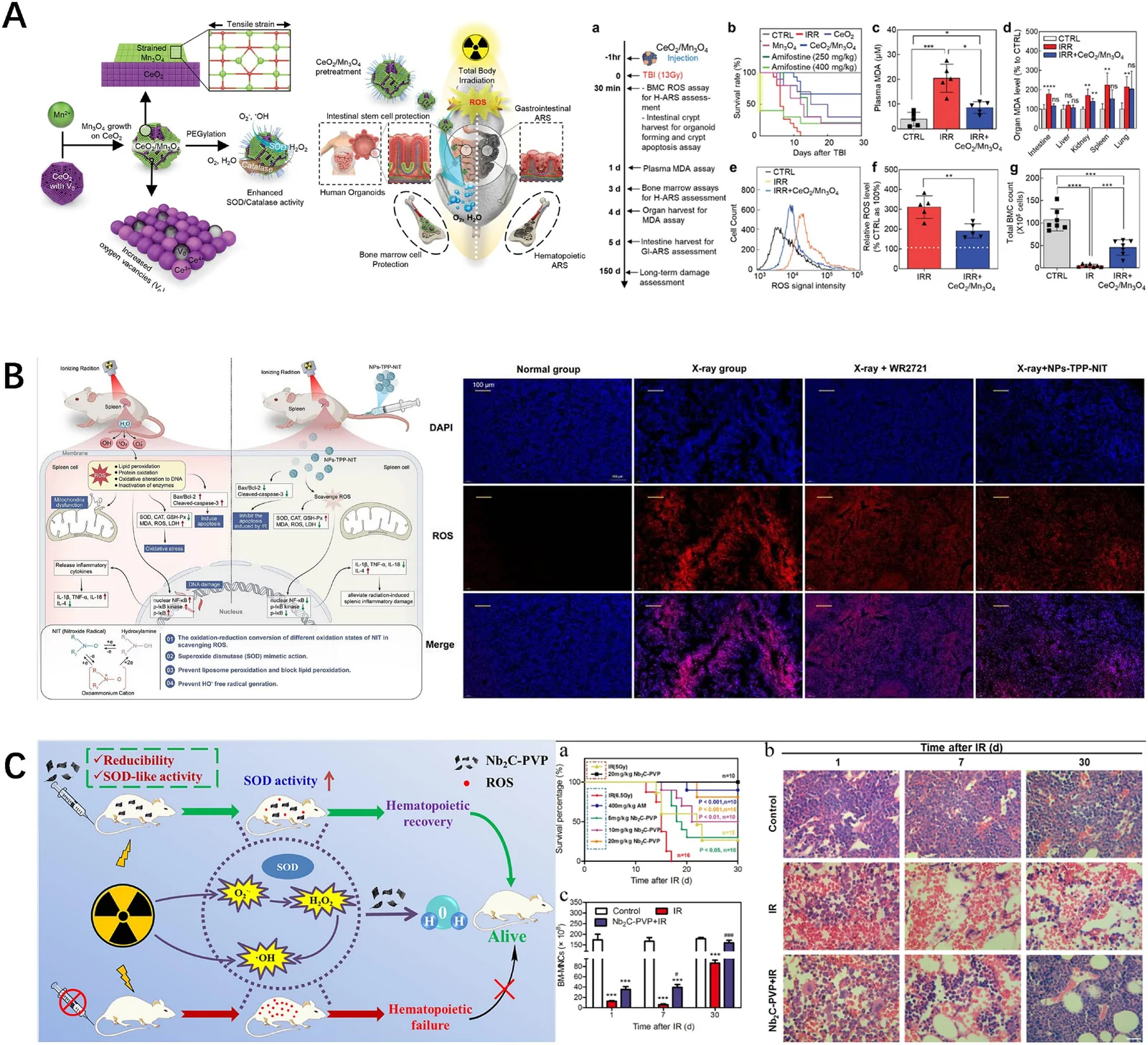
Nanomaterials for suppressing radiation-induced oxidative stress. A) CeO_2_/Mn_3_O_4_ nanomaterials inhibit the generation of ROS in the gastrointestinal tract and bone marrow after radiotherapy. Reproduced with permission from Ref. (Han et al., 2020) Copyright ©2020 Wiley-VCH GmbH. B) NPs-TPP-NIT enhances the activities of superoxide dismutase, catalase, and glutathione peroxidase, thereby protecting immune organs from radiation-induced damage. Reproduced from Ref. (Huang et al., 2024) under the terms of the Creative Commons Attribution-NonCommercial-NoDerivatives (CC BY-NC-ND) 4.0 International License. C) Nb₂C-PVP MXene promotes hematopoietic recovery after radiation by scavenging free radicals and reducing radiation-induced oxidative stress. Reproduced from Ref. (Ren et al., 2019) Copyright ©2019 American Chemical Society.

Cerium oxide nanoparticles (CeO_2_ NPs) represent an important class of redox-active nanozymes for regulating radiation-associated oxidative stress. Their enzyme-mimicking and antioxidant properties enable catalytic regulation of ROS, making them attractive for sustained redox modulation. Recent studies further suggest that integration with polymeric matrices may improve the therapeutic adaptability of nanoceria. For example, incorporation of CeO_2_ NPs into electrospun poly(ε-caprolactone) (PCL) fibers reduced the cytotoxicity associated with direct nanoparticle exposure while retaining ROS-scavenging activity and improving cell survival, indicating that polymer encapsulation may enhance the biocompatibility of CeO_2_-based antioxidant systems (Jahan et al., 2024). The CeO_2_/Mn_3_O_4_ nanocrystals function as catalytic antioxidants, offering protection for the gastrointestinal tract and bone marrow stem cells against ROS generated by radiation exposure. The underlying mechanism involves manganese ions deposited on the surface of CeO_2_ nanocrystals, which form strained layers of manganese oxide (Mn_3_O_4_) islands that enhance the presence of oxygen vacancies. Even at low concentrations, these nanocrystals can mitigate acute radiation syndrome and significantly improve the survival rate of mice subjected to lethal total body irradiation (Fig. 2A).

Importantly, the redox activity of CeO_2_-based nanozymes does not necessarily result in radioprotection and can also be exploited for tumor radiosensitization. Liu et al. developed a multifunctional C/Q@ZIF-8-HA nanoplatform in which CeO_2_-mediated decomposition of H_2_O_2_ increased oxygen availability and alleviated tumor hypoxia, while the integrated system enhanced radiation-induced DNA damage and improved radiotherapeutic efficacy in NSCLC models (Liu et al., 2024). This context-dependent behavior suggests that CeO_2_ may exert either protective or radiosensitizing effects depending on its formulation, localization, and surrounding redox microenvironment. Therefore, polymer encapsulation, surface functionalization, and tumor-targeted delivery may be critical for maximizing normal-tissue protection while avoiding unintended protection of tumor cells. Although functionalized CeO_2_ NPs show promising antioxidant, anti-inflammatory, and imaging properties, uncertainties regarding physicochemical-dependent toxicity, long-term biological fate, and safety remain important barriers to clinical translation (Liu et al., 2024).

Poly (vinylpyrrolidone)-modified MnO_2_ nanoparticles (PVP-MnO_2_ NPs) possessed outstanding stability and multiple free radical scavenging activities. PVP-MnO_2_ NPs not only prevented radiation-induced damage to peripheral blood cells and organs but also improved the survival rate of irradiated mice (Zhou et al., 2023). The alloy of platinum, palladium, and rhodium (PtPdRh) was designed as a hollow structure and protected by polyvinylpyrrolidone for radiation protection. Hollow PtPdRh nanocubes showed remarkable catalytic efficiency in H_2_O_2_ decomposition, meanwhile functioning as SOD-mimetic nanozyme to eliminate superoxide radicals, protecting healthy tissues from ROS-induced injuries (Wang et al., 2018).

As reported, in addition to metallic materials, metal-derived nanoparticles have also found numerous applications in addressing radiation-induced oxidative stress. Pretreatment with PVP-functionalized Nb_2_C nanosheets (Nb_2_C-PVP NS) significantly reduced ionizing radiation-induced ROS, leading to enhanced cell viability in vitro. Moreover, the nanosheets enhanced the activities of superoxide dismutases (SODs) and decreased malondialdehyde (MDA) levels, thus reducing IR-induced pathological damage in the healthy tissues **(**Fig. 2B**)**. In vivo preclinical studies have shown that the survival fraction of MoS_2_-treated mice can be significantly increased to 79% when exposed to high-energy ionizing radiation. In addition, MoS2 spots can help scavenge accumulated free radicals in the body, repair DNA damage, and restore the important chemical and biochemical indices (Zhang et al., 2016).

Au-MoS_2_ also showed significant radioprotective effects in vitro and significantly reduced excess radiation-induced harmful ROS. It rescued radiation-induced DNA damage and protected the bone marrow hematopoietic system from ionizing radiation (Bian et al., 2018). Bi_2_Se_3_NPs can help restore radiation-decreased red blood cell counts, white blood cell counts, and platelet levels. Further studies have shown that Bi_2_Se_3_NPs have free radical scavenging properties and can significantly reduce the levels of malondialdehyde (MDA) (Zhang et al., 2017). MgH_2_ nanomaterials for hydrogen storage and release have the advantage of good stability, high hydrogen storage capacity, and smooth hydrogen release ability. Exposure of MgH_2_ nanomaterials to radiation has been demonstrated to prompt the release of hydrogen and facilitate the scavenging of OH radicals (Ma et al., 2022). MgH_2_ notably enhanced radiation-induced sperm viability and semen quality, playing a pivotal role in safeguarding testicular structure and preserving hormone secretion. The protective effects of MgH_2_ against radiation damage in the male reproductive system are largely attributable to its capacity to mitigate oxidative stress, apoptosis, and cell cycle arrest, thereby ensuring the system's integrity and functionality (Ma et al., 2022).

Triphenylphosphine (TPP) is a lipid-soluble cation with hydrophobic properties that can target mitochondria with negative membrane potential (Yang et al., 2024a). The TPP-modified albumin‑cerium oxide nanoclusters (TPP-PCNLs) abrogated oxidative stress by targeting mitochondria and sustainedly scavenging free radicals. Notably, TPP-PCNLs were able to protect cells from radiation damage and maintain mitochondrial function and morphology. TPP-PCNLs were mainly accumulated in radiation-sensitive tissues, such as liver and spleen, increasing the survival rate of irradiated mice and improving hematopoietic recovery (Yang et al., 2024a). Polyethylene glycol (PEG) modification on nanomaterials could extend the half-life, reduce immunogenicity, and enhance biological stability. PEG-modified cerium dioxide nanoparticles (CNPs) exhibited good protection against radiation damage in human hepatocytes because PEG modification improved the chemical stability and reduced the biotoxicity of CNPs (Li et al., 2015).

Metallofullerenols have recently emerged as another class of potent ROS-scavenging nanomaterials for radioprotection. Their radioprotective activity is closely associated with the ability of the hydroxylated fullerene cage to intercept reactive species generated during water radiolysis. Recent pulse-radiolysis studies demonstrated that Sc_3_N@C_80_(OH)_18_, Lu₃N@C_80_(OH)_18_, and Gd@C_82_(OH)_22_ efficiently react with radiation-derived radicals, providing direct kinetic evidence for their radical-scavenging activity (Grebowski et al., 2023). This antioxidant effect can further limit secondary oxidative injury: Gd@C_82_(OH)_22_ was shown to preserve erythrocyte membrane integrity by suppressing lipid and protein oxidation under oxidative stress (Remigante et al., 2025). More importantly, Sc₃N@C_80_(OH)_18_ directly protected human erythrocytes against γ-radiation-induced biochemical damage and markedly reduced intracellular ROS, indicating that metallofullerenols may act against both primary radiolytic species and subsequent oxidative reactions (Grebowski et al., 2026).

#### 2.2.2 Nanomaterials for enhancing intrinsic antioxidant properties

Beyond directly scavenging excessive ROS in normal tissues to inhibit oxidative stress and alleviate damage, some bioactive nanomaterials can promote the intrinsic antioxidant defense, thereby helping tissues combat oxidative stress to achieve protective effects (Guo et al., 2023). Water-soluble fullerene derivatives with generalized formulas C_60_(OH)_n_ exhibit excellent antioxidant properties due to their hydroxyl groups and are considered highly promising radioprotective agents during infrared radiation damage. They could not only effectively eliminate free radicals and reduce lipid peroxidation, but also maintain redox balance in the body by stimulating the activity of antioxidant enzymes (Biswas et al., 2023). The hydrophilicity and outstanding free radical scavenging ability of fullerenols make them potent alternatives to conventional pharmacological approaches in current radiobiology. A fullerol (C_60_(OH)_36_) protected human erythrocyte membranes and reduced radiation-induced hemolysis under high-energy electron radiation, which was mainly attributed to the function in scavenging ROS (Grebowski et al., 2018). As early as 2008, fullerol (C_60_(OH)_24_) was found to have a protective effect on glutathione peroxidase and glutathione reductase, which positively affected human erythrocyte leukemia cell viability and colony-forming ability after X-ray irradiation (Bogdanović et al., 2008). Radiation led to a decrease in the number of thiol groups in erythrocytes, which was significantly mitigated by C_60_(OH)_36_ by protecting the activities of glutathione peroxidase and glutathione reductase (Grebowski et al., 2022).

Naturally original nanomaterials and their derivatives demonstrate promising antioxidant properties, drawing growing interest for radiation protection. Caffeic acid phenethyl ester (CAPE), a polyphenolic compound derived from propolis, possesses a variety of biological activities such as antioxidant, antiallergic, anticancer, anti-inflammatory, and immunomodulatory, which could increase the activities of superoxide dismutase and glutathione (Chu et al., 2015). CAPE-conjugated nanoparticles showed radioprotection by preventing radiation-induced cell death and ROS accumulation, ultimately increasing survival rate in vivo (Choi et al., 2021). Phytohemagglutinin (PHA-L) is a lectin extracted from red kidney beans, composed of four L-type subunits held together by noncovalent interactions. The PHA-L-derived nanoparticles exerted protective effects on the hematopoietic system and the gastrointestinal tract from radiation damage by activating toll-like receptor 5 (TLR5) (Long et al., 2019). Many nanomaterials can regulate the cellular signaling pathways to exert health-preserving activity. The nanomaterials of triphenylphosphine cation NIT radicals (NPs-TPP-NIT) could enhance the activity of superoxide dismutase, catalase, and glutathione peroxidase, while protecting immune organs from radiation-induced damage by inhibiting the activation of the IκB kinase (IKK)/inhibitor of NF-κB (IκB)/nuclear factor kappa B (NF-κB) signaling pathway in the spleen after irradiation, without compromising the efficacy of radiotherapy **(**Fig. 2B**)**. Recently, probiotics have been demonstrated to play a certain role in radiation protection. A system review summarized that supplementing probiotics can alleviate gastrointestinal damage caused by radiation, including clinical trials in humans. The observed protective effects occur through multiple mechanisms, including antioxidant activity, regulation of immune responses, and preservation of intestinal barrier function (Acharya et al., 2024). The main mechanisms may be accounted for reducing oxidative stress, maintaining intestinal barrier integrity, exerting immunomodulatory effects, producing inhibitory agents, repairing intestinal permeability, upregulating electrolyte absorption in the intestine, and modulating the gut microbiota-metabolite-barrier axis by bacterial supplements (Zheng et al., 2026; Tegegne and Kebede, 2022). At the same time, Ehghaghi et al. found that administering probiotics to experimental mice could reduce the radiation damage caused by X-rays. Moreover, probiotics combined with selenium nanoparticles could further enhance the radiation protection ability by increasing the expression of catalase, SOD, and Catsper genes (Ehghaghi et al., 2022). Lactobacilli probiotics have been proven as a natural radioprotector to protect radiosensitive tissue from damage (Ciorba et al., 2012), and they could also protect intestinal epithelium from radiation injury in a TLR-2/cyclo‑oxygenase-2-dependent manner (Ciorba et al., 2012; Hamade et al., 2022). Fan et al. developed an oral pH-responsive probiotic–liposome hybrid system (EcNPIN-L), which can effectively resist gastric acid and release bacteria under intestinal pH conditions. This nanomaterial reduces pro-inflammatory cytokines (TNF-α, IL-17 A), enhances intestinal barrier function, inhibits epithelial cell apoptosis, and alleviates radiation-induced intestinal injury (Fan et al., 2025).

### 2.3 Nanomaterials with Anti-Inflammatory Effects

Inflammation is the immune system's response to harmful stimuli, designed to protect the body from damage (Zhou et al., 2024). However, overproduced ROS induced by radiation triggers DNA damage and cell death, which activates excessive inflammatory responses (Chen et al., 2023). The inflammatory responses further promote the ROS production, intensifying injury and inflammation, thereby forming a vicious cycle of ROS-inflammatory response loop. Therefore, targeting cellular inflammatory pathways and blocking this vicious cycle using bioactive nanomaterials would be a promising way to prevent radiation-induced damage (Rasoul et al., 2018).

#### 2.3.1 Regulation of inflammatory factors

Selenium nanoparticles (SeNPs) have been found to have anti-inflammatory activity. SeNPs administration in rats can reduce the counts of white blood cells, and levels of tumor necrosis factor-α (TNF-α), prostaglandin E2 (PGE2), thiobarbituric acid reactive substance (TBAR), and total nitrate/nitrite (NOx) in the paw exudate after radiation induction (El-Ghazaly et al., 2017). NPs-TPP-NIT can also protect the immune organs of rats from radiation by blocking the IKK/IκB/NF-κB signaling and reducing the production of IL-1β, TNF-α, IL-18, and IL-4 elevated by X-rays (Huang et al., 2024).

**Fig. 3:**
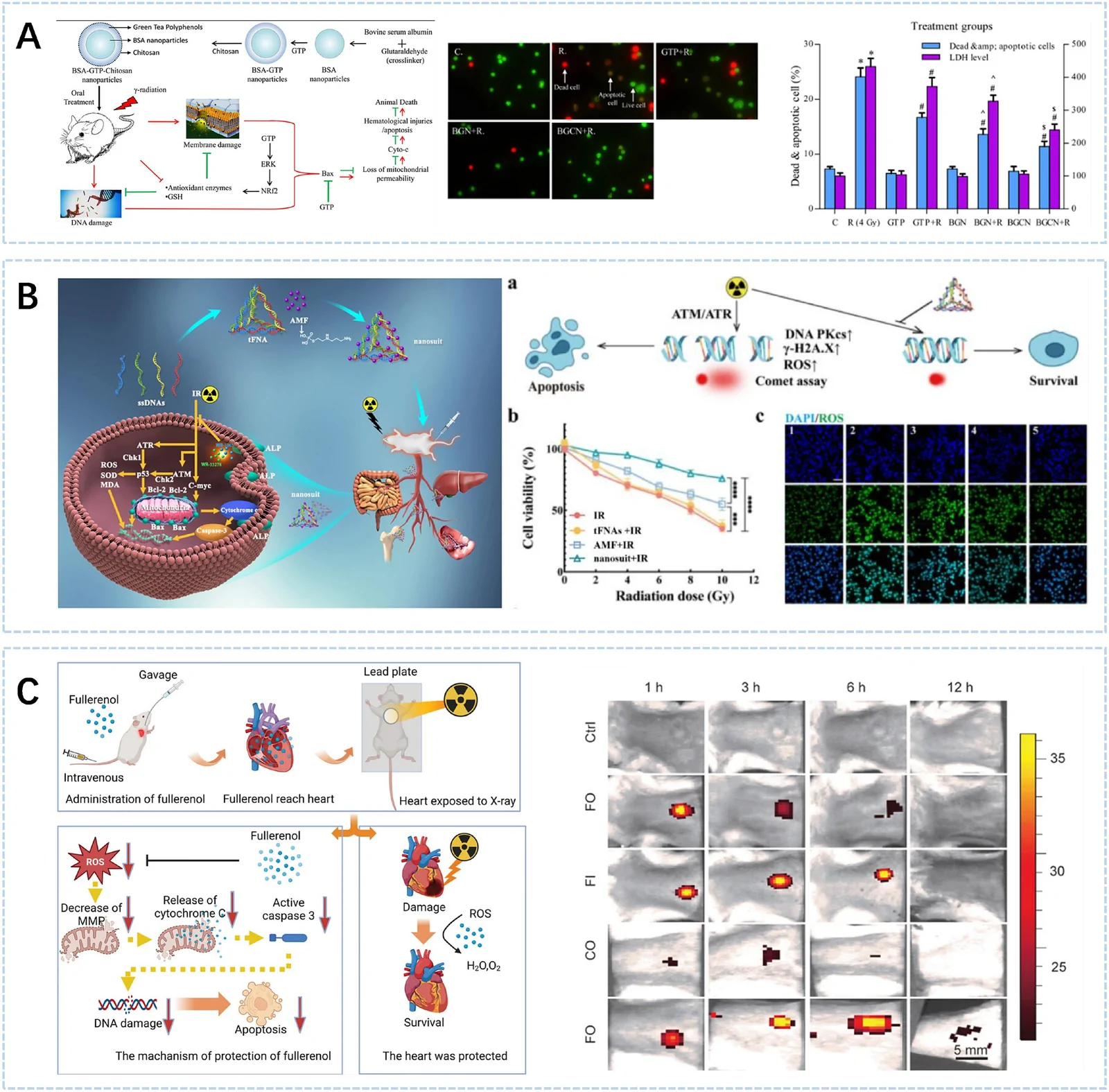
Bioactive nanomaterials targeting cellular inflammatory pathways to prevent radiation-induced damage. A) BSA-GTP-Chitosan nanoparticles mitigate radiation-induced damage by regulating cell apoptosis. Reproduced with permission from Ref. (Kumar et al., 2016) Copyright ©2016 American Chemical Society. B) AMF-loaded tFNAs exhibit enhanced radioprotective effects by inhibiting the ATM/ATR signaling pathway, reducing cell apoptosis, and maintaining redox homeostasis through increased superoxide dismutase (SOD) activity. Reproduced from Ref. (Yang et al., 2024b) under the terms of the Creative Commons Attribution-NonCommercial-NoDerivatives (CC BY-NC-ND) 4.0 International License. C) In cardiomyocytes, fullerene nanoparticles effectively alleviate radiation-induced damage by reducing excessive reactive oxygen species production and inhibiting apoptosis. Reproduced with permission from Ref. (Zhang et al., 2023a) Copyright ©2023 Wiley-VCH GmbH.

Numerous natural compounds have biological activities such as anti-inflammatory and antioxidant, and their nano-formulations have been reported to inhibit radiation-induced apoptosis and reduce the production of cytokines. Oxidized chondroitin sulfate, as a polysaccharide-like component of the extracellular matrix (ECM), was introduced in a multifunctional glycopeptide hydrogel (oCP@As), which can regulate the inflammatory response and accelerate the repair of chronic inflammatory injury by adsorbing inflammatory factors (Guo et al., 2024a). Tea polyphenols exerted radioprotective effects by restoring the redox balance and reducing the expression of Bax, thereby mitigating radiation-induced oxidative damage and apoptosis. Encapsulating tea polyphenols using bovine serum albumin (BSA) as the core matrix and chitosan as the outer shell can further enhance their bioavailability **(**Fig. 3A**)**. Moreover, a synthetic melanin-like polymer, polydopamine nanoparticles (PDA-NPs), demonstrated significant potential in scavenging ROS and inhibiting inflammatory responses. In a murine model, oral administration of PDA-NPs markedly attenuated ionizing radiation-induced DNA damage and enterocyte apoptosis, and significantly suppressed inflammatory responses (Jia et al., 2022). In addition, the natural compounds could be loaded in nanoparticles for radioprotection. Curcumin-loaded chitosan nanoparticles reduced liver injury in rats under radiation and effectively lowered inflammation, liver necrosis, and fibrosis (Azmoonfar et al., 2025).

#### 2.3.2 Anti-apoptosis

Amifostine (AMF) and its active ingredient WR-1065 play anti-apoptosis roles in radiation protection. The tetrahedral framework nucleic acids (tFNAs) were employed as an AMF delivery system, which demonstrated enhanced radioprotection effects by inhibiting ataxia telangiectasia mutated (ATM)/ATM- and Rad3-related (ATR) signaling, reducing cell apoptosis, and maintaining redox homeostasis through increasing SOD activity. In the total body irradiation (TBI) model simulating radiation accidents and the Lewis lung cancer radiotherapy model, the AMF-loaded tFNAs markedly enhanced survival against lethal irradiation, recovered the hematopoietic function, and prevented multi-organ injury. Importantly, this nanosystem selectively protected healthy tissues but not compromising radiotherapeutic efficacy against tumors **(**Fig. 3B**)**. Similarly, WR-1065-loaded nanoscale metal-organic frameworks (MIL-101-Fe) could ameliorate hematopoietic stem cell hematopoietic system damage by inhibiting hematopoietic cell apoptosis, down-regulating BAX and BAK expression, and up-regulating BCL-CL expression (Cao et al., 2020). Besides, the direct interference of apoptotic molecules by nanoparticles can reduce the apoptosis of healthy cells. The incorporation of small interfering RNA (siRNA)/nanoparticle complexes specifically targeting the *PKCδ* or *Bax* genes effectively inhibited the expression of pro-apoptotic proteins, leading to a marked reduction in radiation-induced cell death (Arany et al., 2012).

Mitoquinol (MitoQ) is a mitochondria-targeting antioxidant that consists of TPP^+^ and ubiquinol. MitoQ was prepared as nanoparticles with hyaluronic acid (HA), which were administered to mice before radiation exposure. This treatment exhibited potent radioprotective effects through multiple mechanisms, including suppression of inflammatory factors (NF-κB, IL-1β, and MPO), modulation of apoptotic pathways by suppressing pro-apoptotic caspase-3 and increasing anti-apoptotic Bcl-2 (Dawoud et al., 2024). Similarly, TPP-PCNLs exerted radiation protection by targeting mitochondria to regulate apoptosis, effectively improving survival and hematopoietic function in irradiated animals (Yang et al., 2024a). In addition to acting as an antioxidant agent, fullerol can also regulate cell apoptosis. In cardiomyocytes, fullerol nanoparticles exhibited excellent biocompatibility and effectively alleviated radiation damage by reducing overproduced ROS and suppressing apoptosis **(**Fig. 3C**)**. The upregulated pro-apoptotic protein of p53 acts on mitochondria, leading to the permeability of the mitochondrial outer membrane, the release of apoptotic factors such as cytochrome C, the activation of the caspase cascade, and the initiation of cell apoptosis. Nanoparticles loaded with WR-1065, using MIL-101(Cr) as a carrier, could prevent radiation-induced hematopoietic injury through the p53-dependent apoptotic pathway (Cao et al., 2021).

#### 2.3.3 Immunomodulatory effects

In addition to directly achieving radiotherapy protection by eliminating ROS and suppressing inflammation, some nanomaterials can alleviate radiotherapy damage and promote the recovery of damaged tissues by regulating immune responses. Polyvinylpyrrolidone and selenocysteine modified Bi_2_Se_3_ nanoparticles (PP-Bi_2_ Se_3_@Sec NPs) exhibited strong X-ray attenuation ability. Moreover, the nanoparticles could release selenium to enhance immune functions by increasing the number of white blood cells and reducing bone marrow DNA inhibition, thereby reducing the side effects of systemic radiation (Du et al., 2017). Studies have shown that pretreatment with fullerene derivative C_(60)_(OH)_(n)_ for two weeks can significantly protect the immune functions of the liver and spleen in mice exposed to gamma rays, enhancing the survival rate (Cai et al., 2010). Treatment with cerium dioxide nanoparticles (CNPs) significantly reduced the incidence of ionizing radiation-induced apoptosis and necrosis in human lymphocytes. Moreover, CNPs significantly reduced ionizing radiation-induced intracellular IL-1β production, suggesting that CNPs may play a protective role against ionizing radiation-induced damage by attenuating pro-inflammatory responses in lymphocytes (Zal et al., 2018).

Collectively, antioxidant nanomaterials can mitigate radiation injury through distinct but complementary mechanisms. Conventional antioxidants generally rely on stoichiometric consumption of ROS, whereas nanozyme-based systems such as cerium oxide may provide regenerative catalytic activity. Metallofullerenols, in contrast, combine rapid interception of radiolytic radicals with suppression of secondary oxidative damage. Selenium-containing nanosystems further offer redox-responsive chemistry and can be incorporated into polymeric or degradable nanostructures. However, the clinical superiority of any single antioxidant mechanism remains unproven, because their efficacy is strongly influenced by biodistribution, persistence, dose, and tissue selectivity.

## 3 Nanomaterials for radiation protection

Extensive nanoparticles have been developed and investigated for radiation protection, demonstrating great potential in drug delivery, radioprotective agents, and drug-material synergistic effects. As drug carriers, nanoparticles can precisely deliver drugs to target tissues, enhancing therapeutic effects while reducing side effects. Some functional nanomaterials can also serve as radiation protectants, reducing the radical damage and facilitating functional recovery in injured tissues. Moreover, the combination of nanomaterials and drugs can produce synergistic effects and enhance the therapeutic outcomes. It will be elaborated on the following aspects.

### 3.1 Nanomaterials as drug carriers

The extensive application of radiotherapy in cancer treatment has created an urgent need for developing effective radioprotectants to preserve healthy tissues from radiation damage, thereby enhancing patient survival quality and therapeutic outcomes. Although traditional small-molecule radioprotective agents (e.g., amifostine) have demonstrated clinical efficacy, their therapeutic potential is significantly constrained by poor aqueous solubility, low bioavailability, non-specific biodistribution, and rapid metabolism. These intrinsic limitations significantly impede their radioprotective potential and clinical translation.

To address these issues, nanotechnology-based strategies have emerged as a pivotal approach in drug delivery. By engineering specific nanocarriers (e.g., liposomes, polymeric nanoparticles, dendrimers, inorganic nanoparticles), drug solubility and stability could be significantly enhanced. Moreover, nanocarriers enable targeted drug delivery. Through passive targeting mechanisms (e.g., the enhanced permeability and retention (EPR) effect) or active targeting strategies (e.g., surface modification with targeting ligands), nanocarriers can preferentially deliver cargos to specific tissues or cells requiring protection, minimizing toxicity to non-target sites. Furthermore, nanoengineered systems facilitate controlled drug release (e.g., stimuli-responsive release htriggered by pH values, enzymes, or radiation itself), enabling sustained or on-demand drug release profiles, which reduce dosing frequency and optimize radioprotective efficacy. Capitalizing on these distinct advantages in precision delivery, controlled release, and enhanced drug performance, nanosized delivery systems have been successfully leveraged in radioprotection and are promising for developing safer and more efficient next-generation radioprotective strategies (Han et al., 2024; Chen et al., 2024a).

Multifunctional nanocarriers can integrate drug loading, active or passive tumor targeting, controlled release, imaging, and radiosensitization within a single platform, thereby improving the spatial and temporal precision of cancer therapy (Zuo et al., 2024). Recent advances include ligand-modified, biomimetic, microneedle-assisted, high-Z, and stimuli-responsive systems, in which drug release or therapeutic activity can be regulated by physiological signals such as pH, temperature, biomolecules, and tumor hypoxia, or by external stimuli such as electrical signals and ultrasound. In radiotherapy, these platforms may increase local radiation energy deposition, ROS generation, and tumor radiosensitivity while enabling the co-delivery of chemotherapeutic agents, radiosensitizers, or imaging probes (Zuo et al., 2024; Mostafavi et al., 2025). However, their clinical translation remains constrained by heterogeneous EPR-dependent accumulation, limited tumor penetration, off-target clearance, insufficient control of drug release, and manufacturing and regulatory challenges (Jackson et al., 2024; Zhao et al., 2024).

#### 3.1.1 Delivering synthetic drugs

Amifostine (AMF), a recognized radioprotective agent approved by the U.S. Food and Drug Administration (FDA), has been widely used in radiation medicine (Ji et al., 2023). AMF can provide selective radiation protection to healthy tissues due to the different levels of alkaline phosphatase (ALP) in normal and tumor tissues. The large amount of ALP in normal tissues converts AMF into WR-1065 with radiation-protective activity (Ji et al., 2023; Eisbruch, 2010). However, the short half-life of AMF leads to limited efficacy and complications (Ji et al., 2023). Consequently, increasing AMF's half-life and improving its bioavailability are crucial issues for effective radioprotection.

**Fig. 4:**
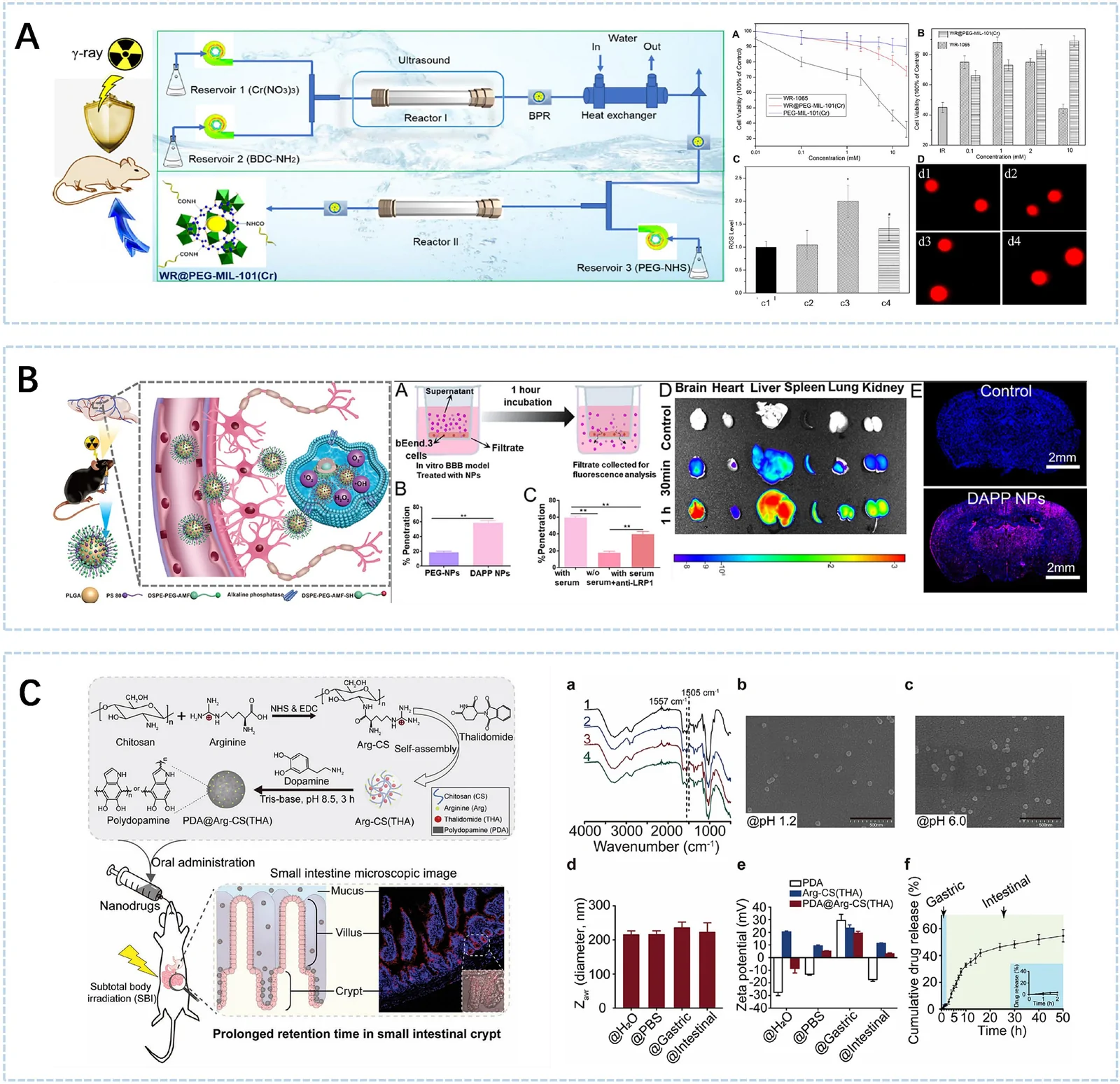
Synthetic drugs combined with nanomaterials exert radioprotective effects during radiotherapy. A) WR@PEG-MIL-101 (Cr) exhibits low toxicity and enhances cell viability by scavenging ROS and inhibiting DNA damage. Reproduced with permission from Ref. (Cao et al., 2021) Copyright © 2021 Elsevier B.V. B) DAPP NPs penetrate the blood–brain barrier, alleviate radiation-induced neural damage and glial cell activation, and provide radioprotection for the brain. Reproduced with permission from Ref. (Zhao et al., 2023) Copyright © 2023 American Chemical Society. C) PDA@Arg-CS(THA) can prolong the intestinal retention time of thalidomide, effectively penetrate into the crypt regions deep within the mucus, improving the survival rate of C57BL/6 J mice under 14 Gy total body irradiation. Reproduced with permission from Ref. (Zhang et al., 2020) Copyright ©2020 Wiley-VCH GmbH.

Chromium-based metal-organic framework (MIL-101 (Cr)) has high porosity and a large specific surface area (up to 3000 m^2^/g), which is regarded as an excellent material for drug delivery. It has good chemical stability and can maintain structural stability in different environments to ensure the safety (Zou et al., 2022). In addition, the surface modification of MIL-101 (Cr) by introducing different functional groups (such as amino groups, PEG, etc.) can enhance the interaction with drug molecules and achieve specific functions (Nikseresht et al., 2024). Therefore, MIL-101 (Cr) is often used as a carrier to achieve targeted delivery and controlled release of drugs. Cao et al. (Cao et al., 2021) prepared PEG-modified MIL-101 (Cr) nanoparticles and loaded with WR-1065 (loading content of 47.2 wt%), referred to as WR@PEG-MIL-101 (Cr). Such nanomedicine exhibited low toxicity and can enhance cell viability by removing ROS and resisting DNA damage **(**Fig. 4A**)**.

To optimize the oral delivery system, researchers utilized the ability of calcium ions to bind with AMF and prepared AMF-loaded calcium carbonate cores (CC/AMF) using a phase transfer co-precipitation method. Furthermore, the cores were modified with phospholipids to obtain hybrid nanoparticles (LCC/AMF). This hybrid material integrated the oral delivery advantages of lipid nanoparticles with the high drug-loading capacity of calcium carbonate, exhibiting excellent stability in aqueous solutions. It facilitated efficient intestinal transport and absorption, enabling significant enhancement of the radioprotective effect of AMF in vivo through oral administration (Wang et al., 2024).

In addition, 1,2-dioleoyl-sn-glycero-3-phosphoethanolamine-N-[poly(ethylene gly*co*l)]-hydroxy was conjugated with AMF and then self-assembled with poly(lactide-*co*-glycolic acid) and polysorbate 80 to form a drug delivery system. This system is capable of penetrating the blood-brain barrier and alleviating radiation-induced neuronal damage and glial cell activation, providing a promising strategy for brain radioprotection **(**Fig. 4B**)**. Liu et al. conjugated curcumin to poly(ethylene glycol) -poly (ε-caprolactone) via a thioketal linker, and generated a ROS-responsive drug carrier for co-loading curcumin and WR-1065. This functional nanodrug reduces the metabolism of curcumin and WR-1065 in the gastrointestinal tract, is efficiently absorbed by cells, and distributed to multiple organs, thereby protecting the hematopoietic system from radiation-induced damage (Liu et al., 2021). DNA-assembled nucleic acid nanomaterials have the advantages of high safety, low biotoxicity, and high transport efficiency. Some researchers used the AMF delivery system built on tFNAs to pretreat irradiated mice. As a result, the content of oxidative stress products in mice was reduced, which not only improved the function of the hematopoietic system but also reduced the radiation damage of other organs and increased the survival rate of mice (Yang et al., 2024b).

Thalidomide was manufactured in 1954 and marketed as a sedative and antiemetic (McFarlane et al., 2018). It has been found to reduce the incidence and severity of radioactive oral mucositis in patients with nasopharyngeal cancer through inhibiting pro-inflammatory cytokine secretion and cell apoptosis through the downstream action factor LZTS3 (Liang et al., 2024). Zhang et al. (Zhang et al., 2020) encapsulated thalidomide into chitosan-based nanoparticles, PDA@Arg-CS(THA), which were then coated with polydopamine. This design endowed it with excellent gastric acid resistance and enabled precisely controlled release with switchable pH in the small intestine environment, significantly enhancing the oral bioavailability of thalidomide as a pyroptosis inhibitor. Experiments in mouse models have shown that these nanomedicines can prolong the residence time in the gut and effectively penetrate the crypt area deep in the mucus. Most importantly, in a C57BL/6 J mouse model treated with 14 Gy of total body irradiation, this nanomedical drug significantly improved the survival of mice and successfully maintained the integrity of their epithelial tissue, demonstrating its potential application in radiotherapy (Fig. 4C).

#### 3.1.2 Delivery of natural compounds

With the revival of traditional Chinese medicine, more and more attention has been paid to the role of Chinese herbs in various diseases. Natural compounds from Chinese herbal medicines have been found to possess anti-inflammatory, antioxidant, and other biological activities, which show potential in radiation protection. However, these natural compounds are often lipophilic with poor water solubility, which leads to low bioavailability and significantly hinders their clinical applications. Therefore, enhancing their bioavailability is an important direction of current research.

**Fig. 5:**
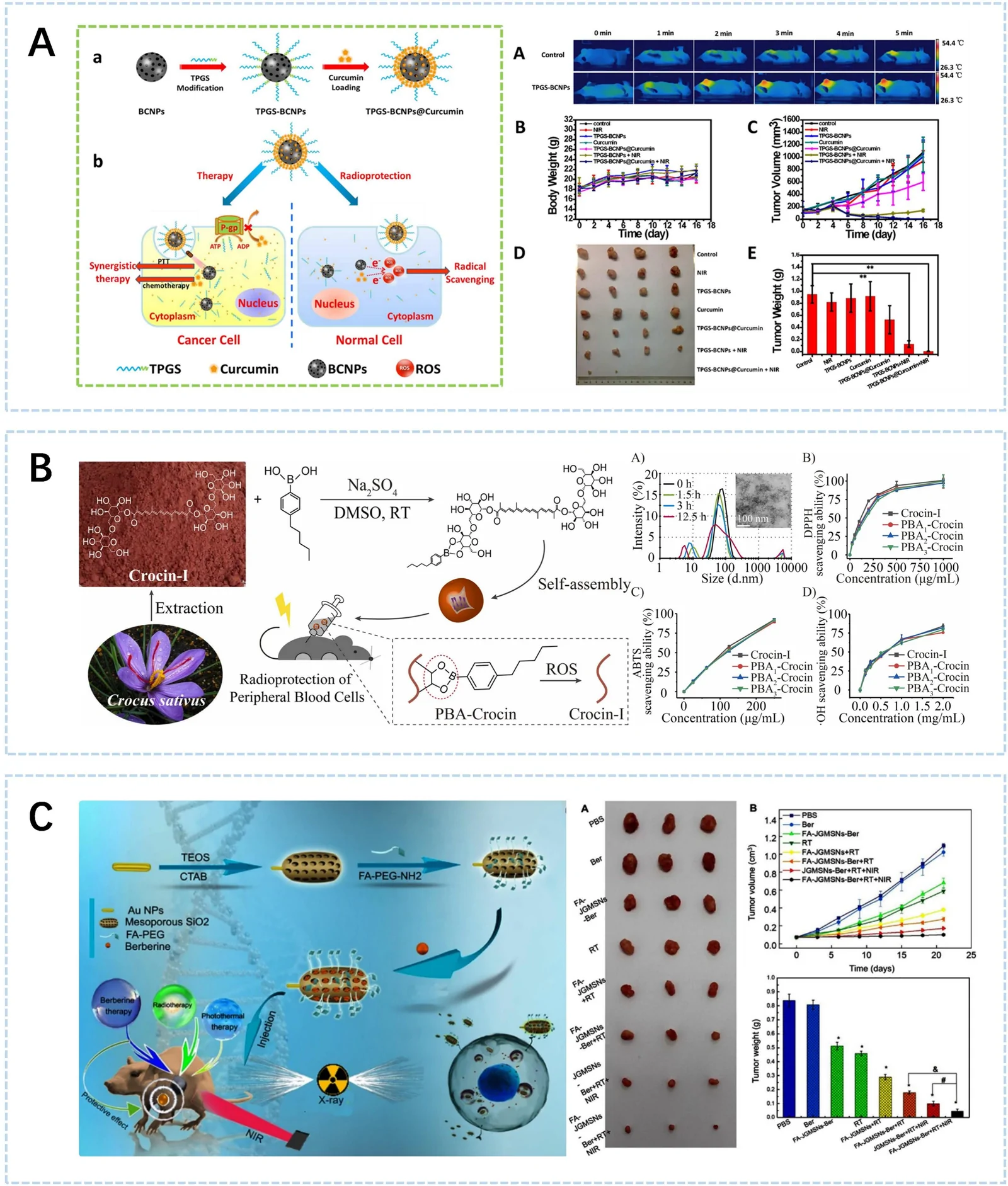
Natural drugs combine with nanomaterials to exert radioprotective effects during radiotherapy. A) TPGS-BCNP@curcumin achieves near-infrared–triggered release of curcumin to enhance antitumor efficacy, meanwhile the complex exhibits strong free radical scavenging activity in normal cells, contributing to its radioprotective effect. Reproduced with permission from Ref. (Xie et al., 2017). Copyright © 2017 American Chemical Society. B) PBA-Crocin is degraded under radiation to release crocin for tissue/cell radioprotection. Reproduced with permission from Ref. (Wang et al., 2022a) Copyright © 2022 Elsevier B.V. C) FA-JGMSNs-Ber can not only act as a radiosensitizer to enhance the efficacy of radiotherapy but also significantly alleviate radiation-induced damage. Reproduced from Ref. (Li et al., 2019) under the Creative Commons Attribution-NonCommercial (CC BY-NC) 3.0 License.

Curcumin, the active ingredient of the spice turmeric rhizome, is a natural polyphenol compound with anti-inflammatory and antioxidant activities, which has been widely investigated in managing diabetes, cardiovascular diseases, and cancer (Kotha and Luthria, 2019). Since curcumin is a potent scavenger of reactive oxygen species (ROS)/nitrogen substances (RNS), this natural molecule is promising for preventing radiation-induced damage (Nelson et al., 2017). To address the limitation of low water solubility and bioavailability, advanced nano-formulations have been developed to deliver curcumin. The “bionic” nano-lipoprotein particles (cNLPs) significantly enhanced curcumin's water solubility (>30-fold) and demonstrated radioprotective efficacy by maintaining sustained antioxidant activity in irradiated AG 05965/MRC-5 human lung fibroblasts (Evans et al., 2022). Xie et al. (Xie et al., 2017) engineered a near-infrared (NIR)-responsive TPGS-BCNP@curcumin for radiation combination therapy. This system achieved dual radioprotection/therapy effects: 1) The bioavailability of curcumin was enhanced by promoting the cellular internalization of TPGS-BCNPs, 2) The TPGS-BCNPs@Curcumin complex enables near-infrared (NIR)-triggered release of curcumin, effectively reducing its efflux and improving its antitumor efficacy, 3) The TPGS-BCNPs@Curcumin complex exhibited strong free radical scavenging activity in normal cells, contributing to its radioprotective effects **(**Fig. 5A**)**. Considering the higher requirements for the stability of the drug and the overall radiation protection effect, a novel polymer, poloxamer188-b-PCL, was designed to be synthesized by ring-opening polymerization of the high-performance polymer PCL with poloxamer 188 to load curcumin (CUR), thereby enhancing the stability of CUR. Poloxamer188-b-PCL nanoparticles exhibited systemic radioprotection potential, with curcumin detected in radiation-sensitive organs, including liver, kidney, and lung. Their sustained free radical scavenging ability significantly reduced radiation-induced lipid peroxidation across multiple tissue types (Lin et al., 2020).

Crocetin, a hydrophilic carotenoid mainly derived from the genus saffron, has been shown to regulate oxidative stress and metabolism, and exhibits anti-inflammatory and anti-nerve damage effects (Boozari and Hosseinzadeh, 2022). However, for crocetin I, its conjugation and glycoside structure make it less stable and have a short half-life, limiting its applications. To address this issue, several studies have been conducted to enhance the stability and anti-cancer efficacy of crocetin-I through nano-capsulation (Soltani et al., 2024). However, few studies have focused on improving its radiation protection. Natural crocin-I was first conjugated with 4-pentylphenylboronic acid (4-PBA) to form PBA-Crocin, which subsequently self-assembled into nanoparticles in an aqueous solution due to its unique amphiphilic structure. The PBA-crocetin nanodrug exhibited great stability and biocompatibility. Of note, due to the ROS-responsive phenylboronic ester linkage, PBA-Crocin could be degraded to release crocin for radioprotection in the tissues/cells under irradiation **(**Fig. 5B**)**.

Baicalein (5, 6, 7-Trihydroxyflavonoids) is a key flavonoid extracted from the dried root of *Scutellaria baicalensis*. Baicalin has a variety of beneficial biological activities, including antioxidant, antibacterial, anti-inflammatory, anticancer, lipid-lowering, anti-atherosclerosis, anti-thrombotic, and immunomodulatory functions (Jang et al., 2019). These characteristics ensure baicalein has great potential and application value in the field of medicine and health care. Joshi et al. (Joshi et al., 2021) successfully prepared and characterized baicalein-loaded solid lipid nanoparticles (SLNB) with efficient drug encapsulation and favorable release kinetics, which demonstrated excellent drug encapsulation efficiency and desirable release kinetics. SLNB showed an obvious protective effect on spleen lymphocyte apoptosis induced by 4 Gy ionizing radiation. When administered orally, SLNB reduced the mortality by ∼70% in mice exposed to full-body irradiation (WBI 7.5 Gy), demonstrating the significant effect of SLNB in radiation protection. Compared to free baicalein, SLNB improved the oral relative bioavailability by approximately 300%. Oral administration of SLNB induced a temporary increase in the number of peripheral blood neutrophils. Notably, when human lung cancer cells (A549) were treated with a radiation protective dose of SLNB, they exhibited enhanced radiosensitivity, as evidenced by reduced cell viability and clonogenesis. In contrast to the antioxidant properties of baicalein in noncancerous cells, SLNB treatment paradoxically increased the ROS levels in cancer cells (A549). This pro-oxidant effect in cancer cells is likely due to: (1) enhanced cellular uptake efficiency of SLNB, and (2) targeted inhibition of thioredoxin reductase (TrxR).

Silymarin is a plant-derived polyphenolic flavonoid mixture derived from the fruit and achene of milk thistle. It consists mainly of three flavonoid lignans: silybin, silydianin, and silychristin. Silymarin has been widely studied and used in medicine and health products because of its remarkable antioxidant, anti-inflammatory, and liver-protective biological activities (Wadhwa et al., 2022). Self-emulsifying drug delivery systems (SNEDDS), formulated from surfactants and cosurfactants, were employed to encapsulate silymarin. Transmission electron microscopy (TEM) showed that the size of silymarin nanoparticles ranged from 3 to 8 nm. The silymarin nanoparticles exhibited an effective protective effect against radiation-induced DNA damage at a concentration of 10 μg/mL in plasmid relaxation tests (Adhikari and Arora, 2015).

Berberine (Ber) is an isoquinoline alkaloid derived from natural plants. Recent studies have shown that berberine exhibits potent anticancer activity while concurrently mitigating radiation-induced organ damage, highlighting its dual therapeutic potential. To enhance the bioavailability of Ber, it was loaded into folate-modified Janus gold mesoporous silica nanocarriers (FA-JGMSNs). FA-JGMSNs-Ber can not only act as a radiosensitizer to enhance the effect of radiotherapy, but also function as a photothermal agent to further enhance the synergistic effect of chemotherapy and radiotherapy through local photothermal therapy. Importantly, FA-JGMSNs-Ber markedly relieved the radiation-induced injury. Thus, FA-JGMSNs-Ber served as both a radiosensitizer to enhance tumor radiotherapy efficacy and a radioprotector to mitigate radiation-induced damage **(**Fig. 5C**)**.

As a non-fermented tea, green tea has won widespread acclaim for its significant health benefits due to its rich polyphenols. Green tea polyphenols (TPs) have been shown to have a variety of benefits, including antioxidant, anti-inflammatory, anti-cancer, and antibacterial (Xing et al., 2019). Using BSA as the matrix and chitosan as the shell, TPs were loaded in nano-formulations with spherical morphology to enhance the stability and maintain the antioxidant activity. The TPs-loaded nanoparticles exhibited excellent radioprotective effects by restoring redox homeostasis via the Nrf2-ERK signaling pathway and suppressing apoptosis through downregulation of pro-apoptotic Bax expression. In a mouse model, orally administered TPs-nanoparticles prior to irradiation significantly mitigated hematological damage to reduce mortality (Kumar et al., 2016).

#### 3.1.3 Delivery of siRNA

Small interfering RNA (siRNA) is a class of double-stranded RNA molecules consisting of 20 to 25 nucleotides that play a key role in regulating gene expression. Its mechanism of action relies on the phenomenon of RNA interference (RNAi), which binds to the target messenger RNA (mRNA), causing the mRNA to break or degrade, ultimately inhibiting the expression of specific genes (Lieberman and Sharp, 2015). siRNA has a wide range of applications, especially in the treatment of diseases. In the radiotherapy of cancer, siRNA can play a synergistic role in enhancing the radiotherapy sensitivity against cancer cells. For example, silencing polo-like kinase 4 (PLK4) in triple-negative breast cancer cells significantly reduced the proliferation of cancer cells during radiotherapy, suggesting the radiotherapy sensitivity of PLK4-targeted siRNA (Pellizzari et al., 2024). Although siRNA has the advantages of high target specificity and long-term performance, which can accurately silence disease-causing genes and reduce side effects, it still faces many challenges in practical applications, such as complex delivery systems, high stability requirements, and difficult cell uptake (Guo et al., 2024b). Notably, engineered nanomaterial-based delivery systems have emerged as a transformative solution to overcome the challenges of siRNA therapeutics. A variety of nanomaterials, including liposomes, polymers, peptides, nucleic acids, and inorganic nanomaterials, have been reported to deliver siRNA as carriers for achieving precise regulation of gene expression (Byun et al., 2023; Çağdaş Tunalı et al., 2023).

A novel pH-responsive nanoparticle was used to deliver functionally active siRNA to salivary gland cell cultures and mouse submaxillary glands. These nanoparticles consisted of cationic micelles that electrostatically complexed with negative-charged siRNA targeting Pkcδ, Bax, and Nkcc1, protected these molecules from nuclease and endosomal lysate components, and finally promoted the release of siRNA into the cytoplasm in the target cells. The expression of proapoptotic protein was significantly inhibited to reduce the radiation-induced apoptotic cells (Arany et al., 2012; Arany et al., 2013). A previous study also explored the role of siRNA nanoparticles in radiation-induced fibrosis complications. They treated model mice with siRNA-loaded chitosan nanoparticles. It was found that the nanoparticles could be taken up by peritoneal macrophages and silence TNF-α in these cells. When these macrophages were recruited to injury sites due to their natural homing effect, the siRNA-loaded nanoparticles silenced the TNF-α expression of macrophages. This suppressed the pro-inflammatory activity of macrophages, thereby exerting a therapeutic effect against fibrosis (Nawroth et al., 2013).

In summary, the nanocarriers discussed in this section have significantly enhanced the radioprotective efficacy of loaded agents by overcoming the key challenges inherent to conventional administration, such as low bioavailability, poor target specificity, and rapid systemic clearance. These engineered delivery systems improve drug stability, enhance penetration across biological barriers, and enable precise delivery and controlled release at the target sites, thereby optimizing therapeutic outcomes while minimizing systemic toxicity. However, the application of nanocarriers in radioprotection still faces challenges, including insufficient understanding of in vivo metabolism, clearance profiles, long-term biosafety, scalable production, and cost-effectiveness.

### 3.2 Radioprotective nanoagents

Extensive research has demonstrated that reducing materials' size to the nanoscale significantly alters their fundamental physicochemical properties. This nanoconfinement effect not only preserves the intrinsic properties derived from the chemical composition but also endows nanomaterials with novel emergent characteristics, such as enhanced catalytic activity, unique surface reactivity, quantum size effects, and distinct biological interactions. These properties offer compelling advantages for diverse applications. This section specifically reviews recent progress in the nanoscale engineering of radioprotective agents.

#### 3.2.1 Carbon-based nanomaterials

In recent years, carbon-based nanomaterials (e.g., fullerenes, graphene, and graphyne) have emerged as promising radioprotective agents, drawing significant research interest due to their good biocompatibility and unique bioactivities. First discovered in 1985, fullerenes appear as closed and hollow cages made entirely of carbon atoms. In the fullerene structure, the carbon atoms are all sp^2^ hybrid and arranged to form 12 pentagons with a certain number of accurately calculated hexagons. When the number of pentagons is kept 12, a simple spherical molecule called C_60_ (outer diameter of ∼0.71 nm) is constructed (Wilson, 1999). The chemical properties of C_60_ are highly similar to those of organic molecules, which has made it attract much attention in the field of chemistry and has become one of the research hotspots. It provided a new research idea and broad application prospects for many directions of new organic materials and drug development. The supramolecular donor-acceptor complex (hydrated C_60_ fullerene C60HyFn) formed by unmodified C_60_ and water molecules showed significant anti-free radical activity in vitro. Intraperitoneal injection of C60HyFn (1 mg/kg) to mice before or after lethal dose X-ray (7 Gy) irradiation increased 30-day survival rates and retarded radiation-induced weight loss (Andrievsky et al., 2009).

However, C_60_ is highly non-polar, exhibiting poor solubility in both water and common organic solvents, which limits its biological applications. Therefore, water-soluble groups or molecules were introduced into the molecular structure of C_60_ to enhance the colloidal stability. For example, carboxyl group-functionalized fullerenes (C_60_(CHCOOH)_2__–__6_), amino-chelated fullerenes (ACFs), and hydroxylated fullerols (C_60_(OH)_n_) have been developed. C_60_(OH)_n_ exhibits tunable aqueous solubility by regulating the modification degree of hydroxyl groups. The water-soluble C_60_ derivatives possess high polarity and electron affinity, not only exhibiting potent ROS trapping ability but also having excellent free radical attachment activity, thereby meeting the requirements for radiation protection. Compared to the clinical standard amifostine (300 mg/kg), fullerenol C_60_(OH)_24_ provided equivalent survival protection against lethal 8 Gy X-ray exposure at the one-third dosage (100 mg/kg), and impressively exhibited higher efficacy in mitigating hematopoietic damage. Notably, C_60_(OH)_24_ exhibited excellent protective effects in radiosensitive organs (e.g, spleen, intestine, and lungs), whereas amifostine showed great shielding effects in the heart, liver, and kidneys. This study suggests that fullerenol is a promising radioprotective nanoagent for specific tissues (Trajković et al., 2007). In another study, fullerenol pretreatment significantly protected irradiated K562 cells by enhancing the antioxidant defense. The superoxide dismutase (SOD) activity and glutathione peroxidase (GPx) levels were significantly promoted by fullerenol, which helped cells resist the radiation-induced oxidative stress (Bogdanović et al., 2008). In mice exposed to a lethal dose of irradiation, C_60_(OH)_24_ pretreatment (2 weeks) effectively reduced the mortality, likely due to reduced oxidative damage and enhanced immune function (Cai et al., 2010). Jacek Grebowski and coworkers synthesized another hydroxylated fullerenol (C_60_(OH)_36_) and found that C_60_(OH)_36_ significantly reduced radiation-induced hemolysis, thiol oxidation, and potassium efflux attributed to its potent antioxidant capability (Grebowski et al., 2018). In addition, C_60_(OH)_36_ enhanced the activities of glutathione peroxidase and glutathione reductase under irradiation, while decreasing glutathione transferase activities, suggesting its potential application for radioprotection (Grebowski et al., 2022).

DF-1 is a mono-branched dendritic C_60_ derivative containing 18 carboxylic groups, which is highly water-soluble and non-toxic. As early as the 1990s, the radiation protective effect of DF-1 was demonstrated in model animals. When DF-1 (100 μmol/L) was administered to zebrafish 3 h pre-irradiation or within 15 min post-irradiation, the radiation toxicity could be significantly alleviated. However, no protective effect was observed when DF-1 treatment was delayed beyond 30 min post-irradiation, suggesting that the protective efficacy of DF-1 is time-dependent (Daroczi et al., 2006). Moreover, the radiation protective efficacy of DF-1 was confirmed in human lymphocytes and rat crypt cells by scavenging cellular ROS and suppressing DNA damage (Theriot et al., 2010).

Graphene is a two-dimensional material with a single-layer sheet structure composed of carbon atoms bonded to a single-layer hexagonal honeycomb lattice by sp^2^ hybridization. It has excellent electrical conductivity, light transmission, and mechanical strength. BSA-modified graphene nanoparticles can remove free radicals in vitro, reduce radiation damage to cellular DNA, and improve cell survival. In vivo experiments revealed that it can protect bone marrow DNA from radiation damage in mice and restore SOD and MDA indicators to normal levels (Xie et al., 2019a). As a graphene derivative, oxidized graphene (GO) has excellent water-soluble, non-toxic, and biodegradable properties, and can also be easily modified. The structure of GO is unique; all carbon atoms are exposed, especially the edge of the carbon atom, and its activity is much higher than that of the planar carbon atom. This open structure allows GO to efficiently trap oxygen radicals on edge carbon atoms. Therefore, the application prospect of GO as a free radical scavenger in the field of radiation protection is highly anticipated. GO (10 μg/mL) scavenging free radicals produced by X-ray radiation protected normal cells from oxidative damage, reduced the levels of DNA damage, and cell death in cells exposed to X-rays (Qiao et al., 2014).

Carbon dots (CDs) represent an emerging class of zero-dimensional carbon nanomaterials (typically smaller than 10 nm). Their core structure can be amorphous carbon, nanocrystalline graphene (or graphene-like structures), or a hybrid state. The surface is typically rich in various oxygen-containing functional groups (e.g., -OH, -COOH, and C=O), and may contain nitrogen-, sulfur-, or other heteroatom-containing groups (Ru et al., 2022; Xia et al., 2019). CDs have demonstrated significant potential in tumor diagnosis and therapy (Kong et al., 2022; Narayanan et al., 2026; Wang et al., 2022b). Recently, nitrogen-doped carbon dots (N-CDs) have been fabricated as a tissue-equivalent dosimeter. These N-CDs exhibit high sensitivity to γ-rays, enabling the detection of ionizing radiation via the oxidation of Fe^2+^ ions to Fe^3+^ ions in Fricke solution (Dos Santos de Almeida et al., 2024), highlighting their potential for radiation warning in healthy tissues.

#### 3.2.2 Selenium-based nanomaterials

The radiation protection effect of selenium-containing compounds was first discovered in 1964 (Shimazu and Tappel, 1964). Selenium compounds can not only provide radiation protection on their own, but also enhance the effects of other radiation protective agents. In practical applications, inorganic selenium compounds (e.g., selenite and selenate), selenium-containing amino acids (such as selenocysteine and selenomethionine), and selenium-containing proteins are widely used. However, these modes of selenium delivery usually have low absorption rates in the gastrointestinal tract and may induce significant toxicity. Therefore, it is particularly important to develop nano-systems that can improve the bioavailability of selenium and facilitate its controlled release in vivo. The nano-sized crystalline selenium particles (SeNPs) were prepared by laser ablation technology, which was less toxic than molecular selenium compounds. SeNPs can significantly improve the viability of cells after radiation exposure and protect proteins and DNA from radiation damage. In the mice receiving radiation, SeNPs reduced chromosome breakage and the number of oxidized proteins, and alleviated radiation-induced leukopenia and thrombocytopenia. SeNPs also normalized gene expression in mouse red bone marrow cells after radiation, demonstrating their role as a novel radiation protector to reduce the harmful effects of ionizing radiation (Gudkov et al., 2023). Wang et al. developed self-assembled selenium-containing polymeric nanoparticles (PVSe) that effectively scavenged radiation-induced ROS while exhibiting reduced toxicity and prolonged blood circulation, thereby enhancing radioprotective efficacy (Wang et al., 2020b). In another study, an increase in lymphocytes and central granulocytes was found in mice after oral SeNPs for one month following different doses of X-rays, suggesting a recovery of bone marrow function (Yazdi et al., 2013). In an animal model of radiation-induced nephropathy, SeNPs were administered intraperitoneally at a dose of 0.1 mg/kg. Impressively, indicators of kidney function were improved, oxidative stress levels were suppressed, and renal pathological features were restored by SeNPs (Karami et al., 2018). Similarly, in cisplatin- and γ-rays-treated rats, SeNPs ameliorate chemoradiation-induced kidney damage due to their antioxidant activity (Saif-Elnasr et al., 2019). SeNPs can also moderate radiation-induced liver injury. Aria et al. found that radiation-induced liver necrosis, fibrosis, and vascular lesions were significantly improved in rats receiving SeNPs (Sohrabi et al., 2020). Selenium nanoparticles can not only play a radiation protection role but also synergistically enhance the protective effect of other radioprotective agents when used in combination. For instance, SeNPs played a regulatory role in limiting X-ray-induced intestinal tissue damage and inflammatory responses, participating in the increase of selenoprotein expression, the reduction of DNA damage responses, and the cyclic GMP–AMP synthase–stimulator of interferon genes (cGAS–STING) pathway cascade reaction. This may provide a reference for the application of Se in the treatment of radiation colitis (Xue et al., 2024). Moreover, SeNPs can synergize with radiotherapy to enhance tumor suppression by inhibiting the expression of proteins related to proliferation, invasion, and migration, stabilizing ROS-induced DNA damage to sensitize cells to apoptosis under ionizing radiation, and enhancing the function of natural killer (NK) cells (Tian et al., 2020; Tang et al., 2023; Gao et al., 2020). Another kind of Se@SiO_2_ NPs was also reported to protect the mouce hippocampus from radiation by inhiting ROS, which might be related to NF-κB and MAPK signaling pathway (Zhu et al., 2022).

#### 3.2.3 Cerium oxide-based nanomaterials

Cerium dioxide nanoparticles (CNPs), as a unique nanozyme, can alternate between Ce^4+^ and Ce^3+^ due to crystal defects, allowing them to mimic the enzymatic activities of antioxidant enzymes (such as SOD and catalase) (Hosseini and Mozafari, 2020). Given the excellent antioxidant capability, CNPs have been widely employed to treat the ROS-related diseases, including neurodegenerative diseases, cardiovascular diseases, inflammatory bowel disease, psoriasis, retinal damage, and cancers (Casals et al., 2020; Li et al., 2023; Xiao et al., 2024). Recently, the radiation protection effects of CNPs have been investigated. In radiobiological studies of MRC-5 and MCF-7 cells, CNPs showed radioprotective effects on normal cells but not on cancer cells (Abdi Goushbolagh et al., 2018). As mentioned earlier, the modified cerium dioxide nanoparticles (CNPs) have good protective effects against radiation damage, likely due to weakening the pro-inflammatory response of lymphocytes (Zal et al., 2018; Park et al., 2008). Another study also investigated the radiation protection capabilities of engineered CNPs. Treating human normal and tumor cells with CNPs and irradiating them with radiation, treatment of normal cells conferred almost 99% protection against radiation-induced death of normal breast epithelial cells, while treatment at the same concentration showed little protection against breast cancer tumor cells (Tarnuzzer et al., 2005). Under the acidic conditions of the tumor microenvironment, the catalase-like activity of CNPs is generally inhibited. This selective enzymatic activity of CNPs may account for the lack of radioprotective effects in tumor cells. Moreover, CNPs also showed their clinical relevance to protect mouse lung tissue from lethal X-ray-induced damage. The protective effect of cerium oxide on radiation-induced stem cell injury was studied in the planaria model. The results showed that nano‑cerium oxide can significantly recover the number of stem cells and enhance the regenerative capacity of tissues. In addition, nano‑cerium oxide was able to reduce cell death and DNA damage caused by low-dose irradiation (Salvetti et al., 2020). In the protective studies on radiation pneumonia, the survival of irradiated mice treated with both low and high doses of nano‑cerium oxide increased by 40%. The structural damage, collagen deposition, inflammatory response, and vascular damage in irradiated mice were significantly alleviated by nano‑cerium oxide (Xu et al., 2016; Colon et al., 2009). In MRC-5 human lung fibroblasts*,* cerium oxide nanoparticles (70 μM) exhibited significant radiation protection and enhanced cell viability against radiation at different doses (100–500 cGy) (Abdi Goushbolagh et al., 2018).

Ionizing radiation can directly interact with mitochondria or indirectly activate the oxidative chain processes, leading to the generation of ROS, which subsequently damages DNA and other cellular structures. Albumin‑cerium oxide nanoclusters (TPP-PCNL) and polyethylene glycol-modified cerium oxide nanoclusters (PCNL) are two types of cerium-based polymeric nanoparticles that can effectively target mitochondria and regulate ROS levels throughout the entire cell. Notably, TPP-PCNLs improved mitochondrial integrity and function in radiation-induced L-02 cells, thereby eliminating persistent damage to nuclear DNA from mitochondrial oxidative stress (Yang et al., 2024a). PCNLs showed superior performance compared to naked CNPs. This is due to the increased Ce^3+^/Ce^4+^ ratio, which enhances oxygen vacancy formation, selectively scavenging ROS generated by ionizing radiation (IR), while also promoting the expression of mitochondrial SOD in cells and effectively reducing DNA damage caused by oxidative stress (Li et al., 2015; Colon et al., 2010).

Although significant progress has been made in the research of various nanomaterials for radioprotection, the field continues to evolve with the emergence of novel catalytically active nanomaterials, commonly referred to as nanozymes. These materials exhibit substantial potential in mitigating radiation-induced damage. In addition to well-established systems, a growing array of innovative nanomaterials is being explored across diverse material classes. Among them, two-dimensional MXenes have attracted considerable attention due to their unique physicochemical properties, including high electrical conductivity, large specific surface area, and tunable surface chemistry. Notably, MXenes exhibit intrinsic multi-enzyme mimetic activities, such as superoxide dismutase (SOD), peroxidase (POD), and catalase (CAT)-like functions, making them promising candidates for scavenging radiation-induced ROS. In addition, nanomaterials derived from natural polymers—such as chitosan, cellulose, hyaluronic acid, and gelatin—offer distinct advantages, including high biocompatibility, biodegradability, and ease of functionalization. These biopolymer-based platforms can be engineered to exhibit intrinsic catalytic activity or serve as carriers for catalytic components, facilitating the development of highly biocompatible nanozymes specifically tailored for radioprotective applications. In addition, innovative hybrid strategies that integrate natural enzymes (e.g., SOD, CAT, glutathione peroxidase [GPx]) with inorganic nanoparticles have emerged as a powerful approach. These hybrid systems leverage the catalytic efficiency and specificity of natural enzymes while benefiting from the enhanced stability, multifunctionality (e.g., targeted delivery), and co-delivery capabilities provided by the nanoparticle component. Such synergistic integration significantly enhances the overall radioprotective performance. Furthermore, the nanozyme repertoire continues to expand with the development of refined formulations of precious metal nanomaterials, novel metal oxides and sulfides, and rationally designed metal-organic frameworks (MOFs). Collectively, these diverse and rapidly advancing catalytic nanomaterials offer unprecedented opportunities for creating next-generation, highly efficient, and targeted radioprotective strategies to mitigate the adverse effects of radiotherapy.

### 3.3 Synergies between drugs and nanomaterials

Beyond serving as delivery vehicles of radioprotective agents or utilizing their intrinsic properties for radiation protection, nanomaterials could also integrate their bioactivity with radioprotective agents to achieve synergistic protective effects. Dhanya et al. first re-dispersed silver nanoparticles (<50 nm) into an aqueous solution of Pluronic F127, and then complexed these nanoparticles with glycyrrhizic acid, an active ingredient from plant cells, to prepare a nanoparticle-glycyrrhizic acid complex. This complex mitigated radiation damage to white blood cells, bone marrow cells, and spleen cells in irradiated mice, improved micronucleus formation, and reduced chromosome aberrations (Chandrasekharan et al., 2011a). Nanoparticle glycyrrheic acid complexes also ameliorated radiation-induced gastrointestinal injury, effectively slowed radiation-induced cellular antioxidant consumption in mouse tissues, and inhibited lipid peroxidation (Chandrasekharan and Nair, 2012). Astaxanthin is a powerful natural antioxidant that could clear radiation-induced ROS and alleviate subsequent DNA damage. Zhang et al. combined astaxanthin nanoparticles (ASXnano) with natural microalgae Spirulina Platensis (SP), a gastrointestinal protective agent, for radiation protection. This complex has the dual advantages of astaxanthin and microalgae Spirulina Platensis. Compared with natural microalgae spirulina Platensis alone, the composite exhibited improved drug solubility, gastric stability, and intestinal absorption. On the other hand, compared with astaxanthin nanoparticles alone, the composite demonstrated enhanced bioavailability and higher drug-loading efficiency (Zhang et al., 2023b). In addition, nanomaterials can also be used in combination with radiotherapy and chemotherapy to synergistically enhance cancer therapy. The Fe_3_O_4_-Au heterodimer coated with BSA possessed radiation protection activity. It was used as a carrier for the co-delivery of radiation sensitizers and chemotherapeutic drugs, which enhanced chemoradiotherapy efficacy against tumors and protected normal tissues (Nosrati et al., 2020). Caffeic acid phenethyl ester (CAPE) has dual effects of radiation sensitization and radiation protection (Taysi et al., 2023). Back in 2017, Nesrin and colleagues administered CAPE to rats treated with total head radiotherapy, aiming to study the radiation protection effects of CAPE. The results showed that the antioxidant state of rat brain tissue was significantly increased, and the level of oxidative stress was significantly decreased. The results suggest that CAPE has a protective effect against brain damage caused by gamma rays (Khayyo et al., 2017). To enhance the water solubility of CAPE and the effect of radiotherapy enhancement, the researchers conjugated CAPE with Pinacyl phenylborate (PBPE) and hyaluronic acid (HA) nanoparticles to form 80–100 nm spherical particles (HAsPBPE). CAPE and CAPE-doped HAsPBPE nanoparticles appropriately prevented radiation-induced cell death and inhibited intracellular accumulation of ROS. As a result, HAsPBPE nanoparticles effectively improved the survival rate of mice from radiation-induced death and reduced apoptosis (Choi et al., 2021). The combination of bioactive nanomaterials and radioprotectants significantly enhances the radioprotection efficiency while reducing side effects, representing a promising research direction. Further exploration of the mechanisms underlying material-drug synergy, along with comprehensive safety studies, will be the key focus of future research and clinical translation.

To provide a concise and critical comparison of the nanomaterial platforms discussed above, their principal radioprotective functions, drug-loading capacity, targeting potential, biosafety, biodegradability, and translational status are summarized in Table 1**.**

**Table 1:** Comparative characteristics and translational considerations of representative nanomaterial platforms for radioprotection.

Nanomaterial platform	Representative systems	Main function	Loading, targeting, and release	Major translational limitation
Polymeric and lipid nanocarriers	LCC/AMF, DAPP NPs, PDA@Arg-CS(THA), cNLPs, SLNB	Improve solubility, stability, bioavailability, and circulation	Moderate–high loading; ligand modification and pH-, ROS-, or NIR-responsive release are feasible	Formulation-dependent toxicity, degradation-product safety, and scale-up
Porous inorganic and hybrid carriers	WR@PEG-MIL-101(Cr), FA-JGMSNs-Ber, Fe₃O₄–Au	High-capacity delivery, imaging, and radiosensitization	High loading; surface functionalization enables targeting and combination therapy	Limited biodegradability, metal retention, and manufacturing complexity
Nucleic acid nanocarriers	tFNA-AMF, pH-responsive siRNA micelles, chitosan–siRNA NPs	Protect nucleic acids and regulate apoptosis- or inflammation-related genes	Sequence-specific targeting; pH-responsive release and cellular delivery	Serum instability, endosomal escape, immune responses, and reproducibility
Redox-active nanoagents	Fullerenols/graphene, SeNPs/PVSe, CeO_2_ nanozymes	Direct radical scavenging, catalytic ROS regulation, and tissue protection	Generally low intrinsic loading; targeting requires further functionalization	Dose-dependent toxicity, uncertain long-term fate, and limited tissue selectivity
Multifunctional synergistic systems	SP@ASXnano, AgNP–glycyrrhizic acid, HAsPBPE	Combine drug delivery with antioxidant, radioprotective, or radiosensitizing effects	Moderate–high loading; multiple therapeutic functions can be integrated	Complex composition, batch consistency, quality control, and regulatory burden

Overall, organic nanocarriers offer greater formulation flexibility and biodegradability, whereas inorganic and redox-active nanomaterials provide high loading capacity or intrinsic ROS-regulating activity. However, no platform currently achieves an optimal balance among targeting efficiency, biosafety, biodegradability, and clinical readiness.

## 4 Prevention and treatment of radiation injury

### 4.1 Radiation-induced skin injury (RISI)

**Fig. 6:**
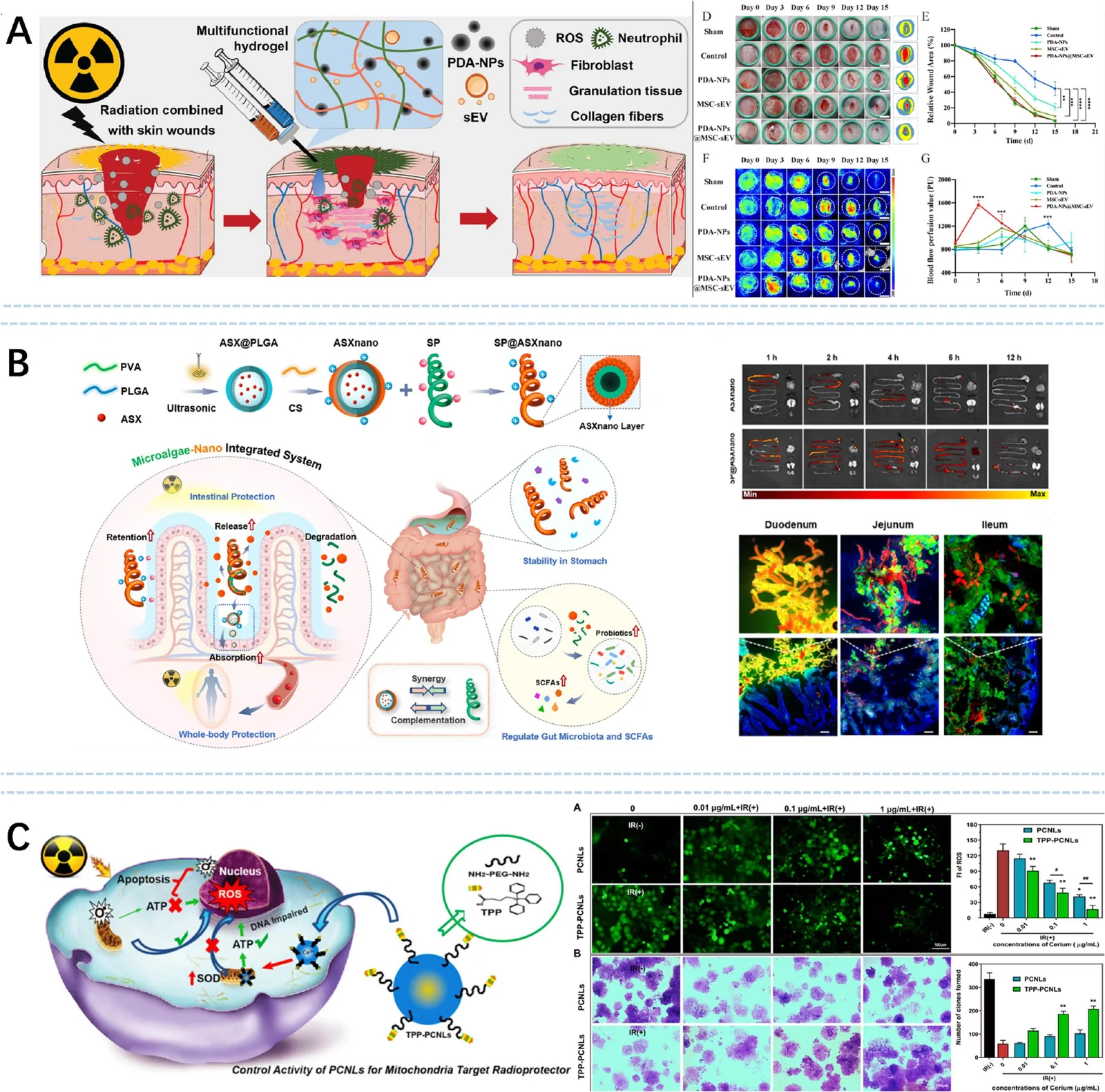
Nanomaterials for the treatment of radiation-induced organ damage. A) PDA-NPs@MSC-sEV hydrogel alleviates radiation-induced skin inflammation and oxidative damage, promotes angiogenesis and cell survival, thereby accelerating healing. Reproduced with permission from Ref. (Fang et al., 2023) Copyright ©2023 Acta Materialia Inc. B) SP@ASXnano synergistically regulates the gut microbiome and lipid metabolism, effectively combating radiation-induced intestinal injury. Reproduced with permission from Ref. (Zhang et al., 2023b) Copyright ©2023 American Chemical Society. Published by Elsevier Ltd. C) TPP-PCNL protects the hematopoietic system by inhibiting ROS. Reproduced from Ref. (Yang et al., 2024a) under the Creative Commons Attribution-NonCommercial (CC BY-NC) 3.0 License.

Skin, the largest organ in the human body, serves critical functions including barrier protection, regulation, immunity, and metabolism, acting as the first line of defense against external damage (Lee and Kim, 2022). Unlike typical burns or ulcers, radiation directly damages epidermal and deeper tissues, resulting in dryness, loss of elasticity, pigmentation, fibrosis, capillary vasodilation, and persistent dermatitis (Kemin and Rutie, 2024). The radiation-induced skin injury (RISI) is often difficult to heal, significantly compromising patients' quality of life. Nanoparticles have emerged as key therapeutic agents for RISI due to their unique radioprotective and regenerative properties (Table 2). To optimize their delivery and therapeutic efficacy, nanoparticles are strategically integrated into hydrogel carriers, leveraging the latter's ability to mimic extracellular matrix (ECM) and provide a conducive wound-healing microenvironment (Su et al., 2022). For example, Fang et al. loaded polydopamine nanoparticles (PDA-NPs) and mesenchymal stem cell-derived small extracellular vesicles (MSC-sEVs) into a Schiff base-crosslinked gelatin/hyaluronic acid hydrogel. These nanoparticles mitigated inflammation and oxidative damage in irradiated skin, enhancing angiogenesis and cell viability to accelerate healing **(**Fig. 6A**)**. In another approach, nano-graphyne was complexed with hyaluronic acid hydrogel, creating a bifunctional radioprotectant. The nanomaterial contributed chemical protection via radical scavenging, while the hydrogel provided physical shielding against low-energy X-rays (Xie et al., 2022). Hao et al. engineered self-assembling antioxidant polypeptides (K16)-based nanoparticles into a heparin-mimicking nanofiber hydrogel. The tyrosine-rich nanopeptides cleared ROS and induced angiogenesis without immune activation, meanwhile, the hydrogel's 3D porous ECM structure accelerated repair. This nanomaterial-hydrogel system suppressed early radiation damage progression and subsequently facilitated epidermal regeneration, inflammation resolution, and tissue remodeling (Hao et al., 2023). Notably, certain nanoparticles like nanoceria function effectively without hydrogels, reducing pigmentation and dermatitis in head/neck radiotherapy (Madero-Visbal et al., 2012).

**Table 2:** Nanomaterials for the prevention and treatment of RISI.

Nanomaterials	Rays	Mechanisms	References
cerium oxide	X-ray 30 Gy	Reducing the incidence of G-III dermatitis and hyperpigmentation of the skin	(Madero-Visbal et al., 2012)
Nano-GDY@SH	X-ray TTS	Shielding low-energy X-rays and removing free radicals	(Xie et al., 2022)
PDA-NPs@MSC-sEV	TTS	Removing ROS	(Fang et al., 2023)
K16	TTS	Removing ROS	(Hao et al., 2023)

TTS: Transdermal Therapeutic System.

### 4.2 Radiation-induced gastrointestinal injury (RIGI)

RIGI is a common complication in radiotherapy, especially in the treatment of pelvic abdominal tumors (Gao et al., 2024). Due to the robust capability of replication and self-renewal, gastrointestinal cells are highly sensitive to radiation. RIGI can cause nausea, vomiting, abdominal pain, and diarrhea in patients, which seriously interferes with tumor treatment and affects the therapeutic outcome (Joshi et al., 2021). The prevention and treatment of RIGI currently present significant challenges. Given the abundance of enzymes (e.g., pepsin and trypsin) in the gastrointestinal tract, orally administered drugs have to possess sufficient environmental tolerance and structural stability to avoid decomposition during gastrointestinal transit (Xie et al., 2019b). Moreover, the drugs are prone to rapid clearance by gastrointestinal fluids, resulting in insufficient accumulation at the injured site and consequently limited therapeutic efficacy (Chen et al., 2024b). To address these issues, functional nanomaterials have been designed to circumvent pharmaceutical efficacy loss caused by gastrointestinal conditions (Table 3).

**Table 3:** Nanomaterials for the prevention and treatment of RIGI.

Nanomaterials	Rays	Mechanisms	References
MitoQ/HA NP, 2 mg/kg/day; IP	γ-ray, whole body, 10 Gy	Removing ROS	(Dawoud et al., 2024)
SP@ASXnano, PO	Whole body	Abilities of anti-inflammation, microbiota protection, and fecal short-chain fatty acid up-regulation	(Zhang et al., 2023b)
PDA-NPs, PO	X-ray, whole body	Suppressing the apoptosis, pyroptosis, and DNA damage of intestinal cells	(Jia et al., 2022)
Poly (ethylene glycol)-poly(ε-caprolactone)	Whole body	Delivering WR-1065	(Liu et al., 2021)
CeO_2_/Mn_3_O_4_	Whole body	Removing ROS	(Han et al., 2020)
GDY-BSA NPs		Inhibiting ROS and reducing apoptosis	(Xie et al., 2020)
C_1_-F_3_P		Preventing radiation-induced lipid peroxidation	(Wu et al., 2020)
2D Nb_2_C MXene, IV	Small intestine, 5/6.5 Gy	Eliminating H_2_O_2_, ^•^OH, and O_2_^•-^	(Ren et al., 2019)
PHA-L Protein	γ-ray, whole body	Removing ROS	(Long et al., 2019)
SN-GLY, PO	γ-ray, whole body, 4 Gy	Reducing the radiation-induced depletion of cellular antioxidants and lipid peroxidation	(Chandrasekharan and Nair, 2012)
CeO_2_, IP	Single-dose radiation	Reducing free radicals and increasing SOD2	(Colon et al., 2010)
DF-1	γ-ray, human lymphocytes and rat intestinal crypt cells	Removing ROS and prevent DNA damage	(Theriot et al., 2010)
WR-1065, PO, 500 mg/kg	γ-ray, whole body, 9 Gy	Removing ROS	(Pamujula et al., 2008)
FNMT, PO, 500 mg/kg	X-ray, whole body, 9 Gy	Removing ROS	(Wang et al., 2022c)

IP: Intraperitoneal injection; PO: per Os; IV: Intravenous.

The abdominal cavity is the primary anatomical compartment for intestinal tissue distribution, which is feasible for drug delivery by intraperitoneal administration. Intrabitoneal injection of mitochondria-targeted mitoquinol/ hyaluronic acid nanoparticles (MitoQ/HA) or cerium oxide can avoid radiation-induced apoptosis of intestinal epithelial cells by scavenging ROS and increasing the activity of antioxidant enzymes (Dawoud et al., 2024; Colon et al., 2010). In addition, Ren et al. (Ren et al., 2019) prepared an ultra-thin 2D NbC MXene and administered it to mice by intravenous infusion, which can reduce the level of radiation-induced malondialdehyde and promote SOD activity, thus achieving radiation protection on multiple organs of the whole body.

The exploration of novel bioactive materials enables precise modification and optimization of oral radioprotective agents. These nanocomposites typically employ stable biomaterials as wrapping shells to protect radioprotective cargos from degradation and denaturation, thereby enhancing the gastrointestinal radioprotective efficacy. Given the complex and harsh conditions of the gastrointestinal (GI) environment—characterized by acidic pH, enzymatic degradation, and mucosal barriers—current research has increasingly focused on optimizing nanomaterial-based drug delivery systems. This optimization is guided by three strategic pillars: rational selection of administration routes, engineering for enhanced GI stability, and strategies to prolong intestinal residence time. Wang et al. (Wang et al., 2022c) developed a nano-montmorillonite-based drug carrier (FNMT) leveraging its excellent intestinal mucoadhesive properties to prolong the retention of fullerenol in the duodenum, thereby mitigating radiation-induced duodenal injury in mice. Moreover, an oral microalgae nanointegration system (SP@ASXnano) based on natural microalgae spirulina obtuse and astaxanthin nanoparticles (ASXnano) was generated to promote intestinal retention and drug availability of ASXnano. SP@ASXnano synergistically modulated intestinal microbiome and lipid metabolism to effectively counteract radiation-induced intestinal damage. SP@ASXNano demonstrates excellent performance in radiation damage protection, including reducing ROS levels and protecting cells throughout the body from radiation damage. In addition, this system also features biological stability and safety, making it suitable for long-term use **(**Fig. 6B**)**.

### 4.3 Radiation-induced hematopoietic injury (RIHI)

The hematopoietic system is extremely sensitive to ionizing radiation, and even moderate doses of ionizing radiation (more than 1 Gy) can trigger acute myelosuppression. Clinical manifestations of this RIHI include bone marrow dysplasia, platelet and neutropenia (Ahmadi et al., 2025). Besides the bone marrow hematopoietic system, radiation also affects the extramedullary hematopoietic organs, including the liver and spleen. Current treatments of RIHI mainly rely on cytokines and stem cells, aiming to reconstruct the hematopoietic system (de Kruijf et al., 2020). However, due to the short half-lives of cytokines and limited efficiency in stem cell transplantation, these approaches exhibited suboptimal therapeutic outcomes. Nanomaterials have been investigated in RIHI radiation protection (Table 4). Albumin‑cerium oxide nanoclusters (TPP-PCNLs) primarily target extramedullary hematopoietic organs, such as the liver and spleen, by modulating mitochondrial apoptosis. It has been demonstrated that TPP-PCNLs effectively improve survival rates, body weight stabilization, and hematopoietic function in vitro (Fig. 6C). Silver nanoparticles (AgNPs) are notable for their unique size-related physicochemical properties and biomedical functions, including their superior antibacterial efficiency and good biocompatibility (Burdușel et al., 2018). Current studies have revealed the antimicrobial mechanisms of AgNPs, including the generation of free radicals, release of silver ions from the particle surface, which in turn trigger cell membrane damage and DNA destruction to inhibit bacterial growth (Dias et al., 2025). Intriguingly, while the anti-bacterial mechanisms of AgNPs are similar to those of radiation-induced tissue damage (e.g., ROS production and DNA damage), emerging evidence incredibly demonstrates the radioprotective effects of AgNPs and AgNPs complexes in the hematopoietic system. Dhanya et al. found that oral administration of AgNPs/glyzyrrhizic acid complex (SN-GLY) could protect leucocytes and bone marrow cells from gamma irradiation by increasing antioxidant enzyme activity and inhibiting chromosomal aberrations (Chandrasekharan et al., 2011a; Chandrasekharan and Nair, 2012). In addition, the silver nanoparticle complex (SN-PAsAG) synthesized by the reduction of vitamin C derivatives could alleviate radiation-induced DNA damage and enhance DNA repair (Chandrasekharan et al., 2011b).

**Table 4:** Nanomaterials for the prevention and treatment of RIHI.

Organs	Nanomaterials	Rays	Mechanisms	References
Extramedullary hematopoietic organs	TPP-PCNL	X-ray, whole body, 4–12 Gy	Removing ROS and protecting mitochondria	(Yang et al., 2024a)
	2D Nb_2_C MXene, IV	Small intestine, 5/6.5 Gy	Eliminating H_2_O_2_, ^•^OH, and O_2_^•-^	(Ren et al., 2019)
	CNPs-AL-PEG	γ-ray, normal liver cells	Elevating SOD2	(Li et al., 2015)
	SN-GLY, PO	γ-ray, whole body, 4 Gy	Reducing the radiation-induced depletion of cellular antioxidants and lipid peroxidation	(Chandrasekharan and Nair, 2012)
	SN-PasAG, PO	Whole body	Repairing cellular DNA	(Chandrasekharan et al., 2011b)
	C_60_(OH)_24_, IP, 40 mg/kg	γ-ray, whole body, lethal dose	enhancing immune function, decreasing oxidative damage, and improving mitochondrial function	(Cai et al., 2010)
	BCL, IP	Whole body, 4/7.5 Gy	Preventing cell apoptosis	(Joshi et al., 2021)
Peripheral blood cell	C_60_(OH)_36_, 150 μg/mL	electron pulses, 650 and 1300 Gy	Protecting antioxidant enzymes	(Grebowski et al., 2022)
	SN-GLY, PO	γ-ray, whole body	Preventing chromosomal aberrations and DNA injury	(Chandrasekharan et al., 2011a)
	PasAG, PO	Whole body	Improving DNA repair rate	(Chandrasekharan et al., 2011b)
	PBA-Crocin, 10 μg/mL		Removing ROS	(Wang et al., 2022a)
Bone marrow hematopoietic tissue	Poly (ethylene glycol)-poly(ε-caprolactone)	Whole body	Delivering WR-1065	(Liu et al., 2021)
	PHA-L Protein	γ-ray, whole body	Removing ROS	(Long et al., 2019)
	Au-MoS_2_		Reducing ROS and DNA injury	(Bian et al., 2018)
	MNPs, IP, 50 mg/kg	γ-ray, whole body, 7 Gy	Preventing DNA damage	(Rageh et al., 2015)
	SN-GLY, PO	γ-ray, whole body, 4 Gy	Reducing the radiation-induced depletion of cellular antioxidants and lipid peroxidation	(Chandrasekharan and Nair, 2012)
	SN-GLY, PO	γ-ray, whole body	Preventing chromosomal aberrations and DNA injury	(Chandrasekharan et al., 2011a)
	PasAG, PO	Whole body	Improving DNA repair rate	(Chandrasekharan et al., 2011b)
	MN, IV	Whole body, 125 cGy	Preventing of free radical formation by melanin	(Schweitzer et al., 2010)
	WR-1065, PO, 500 mg/kg	γ-ray, whole body, 9 Gy	Removing ROS	(Pamujula et al., 2008)
	tFNAs@AMF, IV	X-ray, whole body, 5/6.5 Gy	Regulating oxidative stress	(Yang et al., 2024b)
	Genistein, IM	γ-ray, whole body, 7 Gy	Decreasing proinflammatory factors	(Ha et al., 2013)

IM: Intramuscular Injection.

Natural nanomaterials have received more and more attention because of their bioactivities and good biocompatibility. Phytohemagglutinin-L (PHA-L) protein with a nanoscale size was extracted from a natural plant. PHA-L protein nanoparticles were demonstrated to regulate the immune response via activating TLR5, and efficiently eliminated ROS in the hematopoietic system and gastrointestinal tract under radiation irradiation (Long et al., 2019). Another promising natural nanomaterial is crocin-I nanoparticles, which demonstrate a remarkable radioprotective effect when modified with 4-PBA. Such nanoformulations not only prevented radiation damage to peripheral blood cells but also increased the retention time of crocin-I in plasma by 1.6 times (Wang et al., 2022a).

Radiation-induced hematopoietic injury (RIHI) arises from the complex damage to hematopoietic stem and progenitor cells (HSPCs), bone marrow microenvironment, and extramedullary sites, primarily driven by DNA damage and oxidative stress. This cascade results in acute bone marrow suppression and long-term hematopoietic insufficiency. Although research on conventional radioprotectors and mitigators remains the mainstream approach, studies involving nanomaterials are increasingly demonstrating promising potential in addressing RIHI. The protective effects of nanomaterials on the hematopoietic system mainly involve extramedullary hematopoietic organs, peripheral blood cells, and bone marrow hematopoietic tissues. Their mitigating effects on RIHI are primarily manifested in the following aspects: (1) scavenging reactive oxygen species (ROS) accumulated in the hematopoietic system; (2) protection of cellular DNA, antioxidant enzymes, and mitochondria; and (3) reduction of radiation-induced lipid peroxidation. In the future, nanomaterials may offer further protection of the hematopoietic system by targeting bone marrow or hematopoietic stem cells, repairing the bone marrow microenvironment, developing long-acting sustained-release nanosystems, and integrating both radioprotection and therapeutic strategies. While the search for effective radioprotectors and mitigators continues, silver nanoparticles (AgNPs) represent an intriguing avenue of research. Their documented antioxidant and anti-inflammatory properties in other contexts suggest potential utility in counteracting the primary drivers of RIHI. However, rigorous investigation is critically needed to: (1) elucidate their specific protective mechanisms within the hematopoietic system; (2) define the optimal nanoparticle parameters (e.g., size, surface charge, functionalization) for efficacy and safety; and (3) evaluate their therapeutic window (prophylactic vs. mitigative administration) in relevant in vivo models of radiation injury.

## 5 Clinical translation and applications

The integration of nanomaterials into radiotherapy has emerged as a transformative strategy to mitigate radiation-induced damage to healthy tissues while enhancing therapeutic efficacy. Recent clinical and preclinical studies highlight the potential of engineered nanosystems in addressing key challenges such as oxidative stress, and systemic toxicity. By enhancing the stability, bioavailability, tissue delivery, and controlled release of radioprotective agents, or by directly regulating radiation-induced oxidative stress and inflammation, nanomaterials may provide more effective protection than conventional radioprotectors.

For instance, polyvinylpyrrolidone-modified selenium nanoparticles (PVP-Se NPs) demonstrate significant radioprotective effects by modulating the NF-κB and MAPK signaling pathways, thereby reducing apoptosis and inflammation in irradiated human umbilical vein endothelial cells (HUVECs) and rat models. These NPs enhance antioxidant enzyme activity, suppress pro-inflammatory cytokines (e.g., IL-1β, IL-6, TNF-α), and improve survival rates, positioning them as promising clinical radioprotectors (Li et al., 2024). Similarly, polymer-lipid manganese dioxide nanoparticles (PLMD NPs) overcome hypoxia-related radioresistance in breast cancer models. By alleviating tumor hypoxia and amplifying immunogenic cell death (ICD), PLMD NPs combined with radiotherapy enhance cytotoxic CD8^+^ T-cell infiltration and reduce immunosuppressive Tregs and CD163^+^ macrophages, ultimately inducing abscopal effects to suppress distant untreated tumors (Lip et al., 2026). Mitochondria-targeted NPs-TPP-NIT also protected the spleen by reducing oxidative stress, apoptosis, and activation of the IKK/IκB/NF-κB pathway, while producing no significant protective effect in irradiated tumor cells or tumor tissues (Huang et al., 2024). More recently, diselenide-bridged hollow mesoporous silica nanoparticles displayed cell-type-dependent effects in colorectal cancer radioimmunotherapy. Their degradation products enhanced antioxidant defenses in normal intestinal epithelial cells while increasing oxidative stress and radiosensitivity in colorectal cancer cells. This platform consequently alleviated intestinal radiation injury without compromising tumor control and further enhanced antitumor immune responses (Zhang et al., 2025).

Despite these encouraging findings, a critical concern is that systemically administered antioxidant or ROS-scavenging nanomaterials may also accumulate in tumors and attenuate radiation-induced oxidative stress and DNA damage, thereby reducing the efficacy of radiotherapy. Therefore, selective protection of normal tissues should be experimentally demonstrated rather than inferred solely from improved normal-cell survival. Potential strategies include local or organ-directed administration, surface modification with normal-tissue-specific ligands, optimization of the interval between nanomaterial administration and irradiation, and stimuli-responsive release based on differences in pH, redox status, enzyme activity, or metabolism between normal and malignant tissues (Stasiłowicz-Krzemień et al., 2024). Future studies should assess normal-tissue protection and tumor responses within the same experimental models, including tumor growth, local control, recurrence, antitumor immunity, and overall survival.

Although nanomaterial-based radioprotection remains predominantly preclinical, the clinical development of other radiotherapy-related nanotechnologies provides useful translational experience. Hafnium oxide nanoparticles, represented by NBTXR3, enhance local radiation energy deposition within tumors and have received CE marking for soft-tissue sarcoma treatment (Anselmo and Mitragotri, 2019). Although NBTXR3 is designed as a tumor-directed radioenhancer rather than a normal-tissue radioprotector, its clinical development demonstrates the feasibility of standardized manufacturing, local administration, and clinical evaluation of nanomaterials in radiotherapy. These experiences may inform the future translation of radioprotective nanoplatforms.

Other preclinical nanoplatforms, including hypoxia-modulating manganese dioxide nanoparticles and pH-responsive metal–organic frameworks, further demonstrate that tumor microenvironment-responsive design can improve tumor radiosensitivity and systemic antitumor immunity (Du et al., 2025; Deng et al., 2023).

Despite these encouraging advances, the clinical translation of nanomaterial-based radioprotection remains limited by uncertainties regarding biodistribution, biodegradation, immune responses, clearance, and long-term toxicity. The in vivo behavior of nanomaterials is strongly influenced by their size, shape, composition, surface charge, colloidal stability, protein-corona formation, and route of administration, which collectively determine biological-barrier penetration, cellular uptake, organ accumulation, and elimination pathways (Havelikar et al., 2024; Ma et al., 2024; Abegunde et al., 2025). In particular, uptake by the mononuclear phagocyte system may result in substantial accumulation in the liver and spleen, whereas surface modification and particle size can alter circulation time and tissue distribution. Therefore, radioprotective efficacy should be evaluated together with quantitative whole-body and organ-level biodistribution rather than being inferred solely from short-term therapeutic outcomes.

Biodegradation and long-term biological persistence also vary considerably among nanomaterial platforms. Biodegradable polymeric and lipid-based carriers may reduce long-term accumulation, but their degradation kinetics and the biological effects of degradation products require systematic assessment. In contrast, some inorganic and carbon-based nanomaterials may degrade slowly or remain in tissues for prolonged periods, increasing the potential for delayed organ toxicity. Nanomaterials may also activate complement pathways, alter immune-cell functions, stimulate inflammatory cytokine release, or induce oxidative stress, particularly after repeated administration (Ma et al., 2024; Fortune et al., 2025). Consequently, future preclinical studies should incorporate degradation-product analysis, excretion and clearance assessment, complement and cytokine profiling, repeated-dose toxicity, and long-term histopathological and organ-function evaluation.

Beyond biological safety, successful clinical translation requires standardized physicochemical characterization, reproducible manufacturing, clinically relevant dosing regimens, batch-to-batch consistency, and clearly defined regulatory criteria. Analyses of nanoparticles evaluated in completed human clinical trials indicate that clinical success depends not only on therapeutic efficacy but also on predictable pharmacokinetics, manageable adverse effects, scalable production, and systematic safety monitoring (Joyce et al., 2024). Therefore, a standardized evaluation framework integrating biodistribution, biodegradation, immunotoxicity, long-term safety, manufacturing quality, and regulatory requirements is essential for advancing radioprotective nanomaterials from preclinical studies to clinical practice.

Overall, the application of radiotherapy protective agents in clinical practice is still under research and exploration at present, and more clinical trials and scientific research are needed to verify their efficacy and safety. Future clinical translation should prioritize biodegradable and scalable nanoplatforms with predictable biodistribution, controllable clearance, acceptable immune compatibility, and minimal long-term accumulation. In addition, clinical development should establish whether these platforms can selectively protect healthy tissues without compromising tumor control or antitumor immune responses. Standardized safety evaluation and well-designed clinical trials will therefore be essential for translating promising radioprotective nanomaterials into clinically applicable interventions.

## 6 Conclusions and perspectives

This review comprehensively examines the application of nanomaterials in radiation protection. While traditional radioprotective agents often face limitations such as systemic toxicity, narrow therapeutic windows, and poor targeting specificity, nanomaterials offer distinct advantages. Their high surface-area-to-volume ratio, tunable physicochemical properties (size, shape, composition, surface charge/chemistry), and capacity for multifunctional engineering enable enhanced radiation shielding, ROS scavenging efficiency, and precise delivery of radioprotective payloads to vulnerable tissues. This represents significant progress beyond conventional approaches. We detailed the fundamental principles underlying radiation damage mitigation and categorized nanomaterials based on their protective mechanisms and applications. Furthermore, we summarized the efficacy of nanomaterials in protecting against radiation-induced damage in critical organ systems, notably the gastrointestinal tract, hematopoietic system, and skin. Therein, biodegradable polymeric and lipid-based nanocarriers, stimuli-responsive delivery systems, and intrinsically active redox-regulating nanoagents appear particularly promising, because they combine controllable delivery, tissue protection, and opportunities for functional modification.

Notwithstanding these advances, the field faces substantial challenges impeding large-scale clinical translation. Key limitations include insufficient understanding of the long-term biodistribution, potential systemic toxicity (heavily influenced by factors like size, composition, charge, and degradation profiles), and reliance on murine models that inadequately recapitulate complex human radiation injury, particularly localized damage. Current research often lacks comprehensive integration of in vitro (cell cultures, organoids, organ-on-a-chip), in silico (molecular docking, dynamic simulations, AI-driven modeling), and in vivo methodologies, hindering holistic efficacy and safety evaluation. The molecular mechanisms, especially for nanomaterials possessing intrinsic radioprotective properties, require deeper elucidation beyond current phenomenological observations. Another major knowledge gap is whether nanomaterials can selectively protect normal tissues without reducing tumor radiosensitivity. Future studies should therefore incorporate paired evaluations of normal-tissue protection and tumor response within the same experimental models.

Overcoming these hurdles demands a concerted, multidisciplinary effort. Future endeavors should converge on several pivotal directions:

1) **Intelligent material innovation:** Material design must prioritize intelligent responsiveness (e.g., to ROS, pH, or enzymes at damage sites) for controlled release and targeted action. Greater emphasis should be placed on biodegradable nanomaterials that remain sufficiently stable during circulation and treatment but undergo predictable degradation and clearance after exerting their radioprotective effects. Optimization should extend beyond single functions towards multi-modal/multi-active nanoplatforms combining radioprotection (e.g., ROS scavenging, DNA repair promotion) with tissue regeneration or imaging capabilities. Leveraging artificial intelligence (AI) and big data analytics can accelerate the prediction and design of novel nanomaterials with optimal properties, bioavailability, and reduced toxicity. Machine learning, deep learning, generative models, and inverse-design approaches may be used to predict physicochemical properties, optimize synthesis parameters, model biodistribution, screen surface modifications, and identify formulations with favorable efficacy and safety profiles (Tiwari et al., 2026).
2) **Advanced evaluation and model systems:** Developing larger animal models reflective of human pathophysiology and utilizing sophisticated ex vivo systems (e.g., complex organoids, multi-organ-chips) will provide more predictive data on protection and toxicity. Evaluation protocols should integrate pharmacokinetics, pharmacodynamics, and comprehensive organ function assessment systematically.
3) **Enhanced safety and mechanism-driven design:** Rigorous, long-term biosafety assessment across diverse biological models is paramount, focusing on organ function, survival outcomes, and clearance pathways. This must be coupled with advanced mechanistic studies utilizing high-throughput omics technologies (proteomics, transcriptomics) to identify specific molecular targets and signaling pathways (e.g., NRF2, NF-κB) modulated by nanomaterials, informing the rational design of safer, more effective agents. The resulting data should be incorporated into safe-by-design frameworks to guide the selection of material composition, particle size, surface chemistry, degradation rate, and dosing regimen before clinical development (Havelikar et al., 2024; Abegunde et al., 2025).
4) **Accelerating clinical translation:** Expanding research focus to understudied radiation-sensitive organs (e.g., eyes, liver, and spleen) and developing personalized nano-delivery strategies based on individual risk profiles are essential. Personalized nanomedicine should integrate patient-specific factors, including tumor location and molecular characteristics, radiation dose distribution, normal-tissue radiosensitivity, immune status, organ function, and nanoparticle pharmacokinetics, to guide nanomaterial selection, administration route, dose, and treatment schedule. AI-assisted modeling may further support patient stratification, prediction of tissue exposure and therapeutic response, and adjustment of nanomaterial formulations for precision radiotherapy (Tiwari et al., 2026; Sheikh and Jirvankar, 2024). A critical translational bottleneck is elucidating the in vivo fate of nanomaterials—balancing stability for function with timely biodegradability and clearance to minimize accumulation risks. Prospective clinical data mining can guide material design towards addressing real-world clinical needs. Bridging materials science, molecular biology, computational science, and clinical medicine is fundamental to refine candidate nano-radioprotectants, enabling their transition from promising laboratory discoveries into clinically viable, high-efficacy, low-toxicity solutions for diverse radiation protection scenarios, including radiotherapy and accidental exposure.
5) **Optimization of clinically applied radioprotective agents:** Currently, the number of radioprotective agents approved for clinical use remains limited, and their applications are relatively narrow in scope. Amifostine, an organic thiophosphate compound, is widely regarded as one of the most effective radioprotective agents available. Its primary mechanism of action involves scavenging free radicals generated during radiotherapy, thereby reducing damage to normal tissues. However, the clinical application of amifostine is constrained by several drawbacks, including potential adverse effects such as hypotension and nausea (Ji et al., 2023). More recently, a medical anti-radiation spray composed of superoxide dismutase, vitamin B12, and lactose has been introduced. This formulation can rapidly and effectively penetrate the skin or wound surfaces to eliminate large amounts of radiation-induced free radicals, thereby attenuating inflammatory responses (Stasiłowicz-Krzemień et al., 2024). While these agents can partially mitigate radiation-induced injury to normal tissues, they are insufficient to fully prevent the side effects associated with radiotherapy. Therefore, future research should focus on further optimizing these clinically approved agents to enhance their protective efficacy and provide more comprehensive support during radiotherapeutic interventions. In parallel, nanomaterial-based radioprotection should be explored in combination with immunotherapy, particularly when nanoformulations can alleviate radiation-induced normal-tissue inflammation while preserving or enhancing antitumor immune responses. A list of chemical compounds discussed in this review is provided at the end of the manuscript.

List of chemical compounds

**Table:** 

No.	Common Name	IUPAC Name	CAS No.
1	Amifostine	2-(3-aminopropylamino)ethylphosphorothioic acid	20,537–88-6
2	WR-1065	2-[(3-aminopropyl)amino]ethanethiol	31,087–87-2
3	Curcumin	(1E,6E)-1,7-bis(4-hydroxy-3-methoxyphenyl)hepta-1,6-diene-3,5-dione	458–37-7
4	Thalidomide	2-(2,6-dioxopiperidin-3-yl)-1H-isoindole-1,3(2H)-dione	50–35-1
5	Baicalein	5,6,7-trihydroxyflavone	491–67-3
6	CAPE	2-phenylethyl (2E)-3-(3,4-dihydroxyphenyl)prop-2-enoate	104,594–70-9
7	Crocin	bis(2,3,4,5,6-pentahydroxyhexosyl) 8,8′-diapo-ψ,ψ-carotenedioate	42,553–65-1
8	Berberine	9,10-dimethoxy-5,6-dihydro[1,3]dioxolo[4,5-*g*]isoquinolino[3,2-a]isoquinolin-7-ium	2086-83-1
9	Cerium dioxide	Cerium(IV) oxide	1306-38-3
10	Fullerenol	Polyhydroxylated C60 fullerene	162,639–62-1

## CRediT authorship contribution statement

**Yizhi Ge:** Writing – original draft, Software, Methodology, Investigation, Formal analysis, Data curation, Conceptualization. **Minghong Xu:** Software, Methodology, Formal analysis, Data curation. **Xingyu Yang:** Validation, Software, Methodology, Data curation. **Wen Yun:** Supervision, Software, Resources, Formal analysis. **Wenxuan Huang:** Writing – review & editing, Supervision, Methodology, Data curation, Conceptualization. **Jia Liu:** Writing – review & editing, Visualization, Supervision, Resources, Project administration, Funding acquisition, Conceptualization.

## Ethics approval

N.A.

## Author contributions

Yizhi Ge and Minghong Xu contributed to the conceptualization, design, writing, methodology, and analyses of this study. Xingyu Yang and Wen Yun contributed to the conception, analysis, and interpretation of data for the work. Wenxuan Huang and Jia Liu performed conceptualization, design, data analysis, and critically revised the manuscript. All authors approved the final version and agreed to be accountable for all aspects of the work.

## Funding

We appreciate the funding support from the 10.13039/501100001809National Natural Science Foundation of China (82472135), the 10.13039/501100003819Natural Science Foundation of Hubei Province (2024AFA053), the 10.13039/501100002949Postdoctoral Fund of People's Government of Jiangsu Province, China (200730000107), and the Key Research Program of the 10.13039/100012554Hubei Provincial Department of Education (D20251405).

## Declaration of competing interest

The authors declare that they have no known competing financial interests or personal relationships that could have appeared to influence the work reported in this paper.

## Data Availability

The custom code used in this study is not publicly available but can be obtained from the corresponding author upon reasonable request. All figures are exhibited under the terms of the Creative Commons Attribution License as permitted Copyright.

## Appendix A Supplementary data

Supplementary material: Supplementary Tables S1–S3. Nanomaterials for the prevention and treatment of radiation-induced gastrointestinal injury (RIGI) and radiation-induced hematopoietic injury (RIHI), together with detailed chemical information on the compounds included in this review.
