# Supplementary material for: Protective mechanisms and therapeutic promise of nanomaterials in radiation-induced diseases
Source: Int J Pharm X. 2026 Sep 3;12:100658. doi: 10.1016/j.ijpx.2026.100658 (PMC13579450; doi:10.1016/j.ijpx.2026.100658)
Supplement: Supplementary file 1 [file mmc1.docx]

**Supplementary Table 1. Nanomaterials for the prevention and treatment of RIGI**

| Nanomaterials | Rays | Mechanisms | References |
| --- | --- | --- | --- |
| MitoQ/HA NP, 2 mg/kg/day; IP | γ-ray, whole body, 10 Gy | Removing ROS | (83) |
| SP@ASXnano, PO | whole body | Abilities of anti-inflammation, microbiota protection, and fecal short-chain fatty acid up-regulation | (163) |
| PDA-NPs, PO | X-ray, whole body | Suppressing the apoptosis, pyroptosis, and DNA damage of intestinal cells | (79) |
| Poly (ethylene glycol)-poly(ε-caprolactone) | whole body | Delivering WR-1065 | (99) |
| CeO_2_/Mn_3_O_4_ | whole body | Removing ROS | (35) |
| GDY-BSA NPs |  | Inhibiting ROS and reducing apoptosis | (40) |
| C_1_-F_3_P |  | Preventing radiation-induced lipid peroxidation | (177) |
| 2D Nb_2_ C MXene, IV | small intestine, 5/6.5 Gy | Eliminating H_2_O_2_, ^•^OH, and O_2_^•-^ | (49) |
| PHA-L Protein | γ-ray, whole body | Removing ROS | (63) |
| SN-GLY, PO | γ-ray, whole body, 4 Gy | Reducing the radiation-induced depletion of cellular antioxidants and lipid peroxidation | (162) |
| CeO_2_, IP | single-dose radiation | Reducing free radicals and increasing SOD2 | (160) |
| DF-1 | γ-ray, human lymphocytes and rat intestinal crypt cells | Removeing ROS and prevent DNA damage | (132) |
| WR-1065, PO, 500 mg/kg | γ-ray, whole body, 9 Gy | Removing ROS | (178) |
| FNMT, PO, 500 mg/kg | X-ray, whole body, 9 Gy | Removing ROS | (176) |

IP: Intraperitoneal injection; PO: per Os; IV: Intravenous.

**Supplementary Table 2. Nanomaterials for the prevention and treatment of RIHI**

| Organs | Nanomaterials | Rays | Mechanisms | References |
| --- | --- | --- | --- | --- |
| Extramedullary hematopoietic organs | TPP-PCNL | X-ray, whole body, 4-12 Gy | Removing ROS and protecting mitochondria | (54) |
|  | 2D Nb_2_ C MXene, IV | small intestine, 5/6.5 Gy | Eliminateing H_2_O_2_, ^•^OH, and O_2_^•-^ | (49) |
|  | CNPs-AL-PEG | γ-ray, normal liver cells | Elevating SOD2 | (53) |
|  | SN-GLY, PO | γ-ray, whole body, 4 Gy | Reducing the radiation-induced depletion of cellular antioxidants and lipid peroxidation | (162) |
|  | SN-PasAG, PO | whole body | Repairing cellular DNA | (183) |
|  | C_60_(OH)_24_, IP, 40 mg/kg | γ-ray, whole body, lethal dose | enhancing immune function, decreasing oxidative damage, and improving mitochondrial function | (129) |
|  | BCL, IP | whole body, 4/7.5 Gy | Preventing cell apoptosis | (112) |
| Peripheral blood cell | C_60_(O)_36_, 150 μg/mL | electron pulses, 650 and 1300 Gy | Protecting antioxidant enzymes | (60) |
|  | SN-GLY, PO | γ-ray, whole body | Preventing chromosomal aberrations and DNA injury | (161) |
|  | PasAG, PO | whole body | Improving DNA repair rate | (183) |
|  | PBA-Crocin, 10 μg/mL |  | Removing ROS | (184) |
| Bone marrow hematopoietic tissue | Poly (ethylene glycol)-poly(ε-caprolactone) | whole body | Delivering WR-1065 | (99) |
|  | PHA-L Protein | γ-ray, whole body | Removing ROS | (63) |
|  | Au-MoS_2_ |  | Reducing ROS and DNA injury | (50) |
|  | MNPs, IP, 50 mg/kg | γ-ray, whole body, 7 Gy | Preventing DNA damage | (185) |
|  | SN-GLY, PO | γ-ray, whole body, 4 Gy | Reducing the radiation-induced depletion of cellular antioxidants and lipid peroxidation | (162) |
|  | SN-GLY, PO | γ-ray, whole body | Preventing chromosomal aberrations and DNA injury | (161) |
|  | PasAG, PO | whole body | Improving DNA repair rate | (183) |
|  | MN, IV | whole body, 125 cGy | Preventing of free radical formation by melanin | (186) |
|  | WR-1065, PO, 500 mg/kg | γ-ray, whole body, 9 Gy | Removing ROS | (178) |
|  | tFNAs@AMF, IV | X-ray, whole body, 5/6.5 Gy | Regulating oxidative stress | (80) |
|  | Genistein, IM | γ-ray, whole body, 7 Gy | Decreasing proinflammatory factors | (187) |

IM: Intramuscular Injection.

**Supplementary Table 3. Detailed Chemical Compounds Table**

| No. | Common Name | IUPAC Name | CAS No. |
| --- | --- | --- | --- |
| 1 | Amifostine | 2-(3-aminopropylamino)ethylphosphorothioic acid | 20537-88-6 |
| 2 | WR-1065 | 2-[(3-aminopropyl)amino]ethanethiol | 31087-87-2 |
| 3 | Curcumin | (1E,6E)-1,7-bis(4-hydroxy-3-methoxyphenyl)hepta-1,6-diene-3,5-dione | 458-37-7 |
| 4 | Thalidomide | 2-(2,6-dioxopiperidin-3-yl)-1H-isoindole-1,3(2H)-dione | 50-35-1 |
| 5 | Baicalein | 5,6,7-trihydroxyflavone | 491-67-3 |
| 6 | CAPE | 2-phenylethyl (2E)-3-(3,4-dihydroxyphenyl)prop-2-enoate | 104594-70-9 |
| 7 | Crocin | bis(2,3,4,5,6-pentahydroxyhexosyl) 8,8'-diapo-ψ,ψ-carotenedioate | 42553-65-1 |
| 8 | Berberine | 9,10-dimethoxy-5,6-dihydro[1,3]dioxolo[4,5-g]isoquinolino[3,2-a]isoquinolin-7-ium | 2086-83-1 |
| 9 | Cerium dioxide | Cerium(IV) oxide | 1306-38-3 |
| 10 | Fullerenol | Polyhydroxylated C60 fullerene | 162639-62-1 |
